# Is evolution faster at ecotones? A test using rates and tempo of diet transitions in Neotropical Sigmodontinae (Rodentia, Cricetidae)

**DOI:** 10.1002/ece3.8476

**Published:** 2021-12-16

**Authors:** André Luís Luza, Renan Maestri, Vanderlei Júlio Debastiani, Bruce D. Patterson, Sandra Maria Hartz, Leandro D. S. Duarte

**Affiliations:** ^1^ Programa de Pós‐Graduação em Ecologia Departamento de Ecologia Instituto de Biociências Universidade Federal do Rio Grande do Sul Bairro Agronomia Rio Grande do Sul CEP 91501‐970 Brazil; ^2^ Departamento de Ecologia e Evolução Universidade Federal de Santa Maria Santa Maria Rio Grande do Sul CEP 97105‐900 Brazil; ^3^ Negaunee Integrative Research Center Field Museum of Natural History Chicago Illinois USA

**Keywords:** ancestral character mapping, ancestral character reconstruction, macroecology, macroevolution, Neotropics, niche evolution

## Abstract

We evaluated whether evolution is faster at ecotones as niche shifts may be needed to persist under unstable environment. We mapped diet evolution along the evolutionary history of 350 sigmodontine species. Mapping was used in three new tip‐based metrics of trait evolution – Transition Rates, Stasis Time, and Last Transition Time – which were spatialized at the assemblage level (aTR, aST, aTL). Assemblages were obtained by superimposing range maps on points located at core and ecotone of the 93 South American ecoregions. Using Linear Mixed Models, we tested whether ecotones have species with more changes from the ancestral diet (higher aTR), have maintained the current diet for a shorter time (lower aST), and have more recent transitions to the current diet (lower aLT) than cores. We found lower aTR, and higher aST and aLT at ecotones than at cores. Although ecotones are more heterogeneous, both environmentally and in relation to selection pressures they exert on organisms, ecotone species change little from the ancestral diet as generalist habits are necessary toward feeding in ephemeral environments. The need to incorporate phylogenetic uncertainty in tip‐based metrics was evident from large uncertainty detected. Our study integrates ecology and evolution by analyzing how fast trait evolution is across space.

## Graphical abstract

We explored whether evolution is faster at ecotone zones by developing three new tip‐based metrics — a species‐specific characterization of trait evolution — and spatializing them at the species‐assemblage level. We showed that evolution's speed — in terms of rates and time of diet transition — varies over space, with ecoregion ecotones having species with fewer transitions from the ancestral diet, longer stasis time of the current diet, and older transition to the current diet than ecoregion cores. Our research provides a formal link between macroecology and macroevolution, which is still rare in the ecological literature.

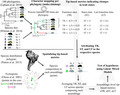

## 1 INTRODUCTION

To disentangle the mechanisms producing the biological diversity seen in nature, ecologists increasingly seek to integrate ecology and evolution (Jetz et al., 2012; McGill et al., 2019; Wiens & Donoghue, 2004). Mapping traits onto phylogenies is essential for such integration, as mapping traits reveals the rates and tempo of evolution of behavioral, morphological, and ecological characteristics (Bollback, 2006). Knowledge of trait evolution has often been applied to evaluate the evolutionary mechanisms producing, for example, the appearance of ecological innovations and the bursts behind evolutionary radiations (Cantalapiedra et al., 2014; Joy et al., 2016; Maestri et al., 2017). Nevertheless, despite extensive study on rates of trait evolution over time and across clades (Gingerich, 2009; Joy et al., 2016 and references therein), understanding how these rates vary over space is equally challenging, and still little understood.

In terms of species diversification, rates are heterogeneous over space. Between‐biome comparisons suggest that some biomes are more speciation‐prone than others (Antonelli et al., 2018; Davies & Buckley, 2011; Goldberg et al., 2005). For example, Amazonian tropical forests were inferred to be the main source of Neotropical biodiversity due to high speciation and low extinction rates, yielding species accumulation within tropical forests (Antonelli et al., 2018; Davies & Buckley, 2011). Marine tropical biomes appear to be sources of temperate‐region bivalves owing to the dispersal of taxa that evolved in tropical regions (Goldberg et al., 2005). Montane portions of the Andes and also of the Atlantic Rainforest were shown to be centers of early rodent diversification and diversity accumulation into the Neotropics (Leite et al., 2014; Maestri et al., 2019). Although these findings implicate cradles and museums of biodiversity, we still need to know the situations where diversification results in trait evolution (Oliveira et al., 2016), as well as the roles of historical and ecological factors in producing spatial heterogeneity in trait evolution.

Here, we tested whether within‐biome heterogeneity in species trait evolution would be related to the distance from spatially and temporally unstable ecotones. If this relationship holds, then assemblage position relative to ecoregion boundary, or its interaction with habitat type, should be the main predictor of evolutionary speed relative to other ecological and historical variables like habitat type, neighborhood characteristics, and location (either in the Andes or Atlantic Rainforest). Ecotone is a concept used at several spatial scales to characterize the boundary between habitat patches; the environmental contrast between adjacent patches can produce boundaries that organisms may perceive (Cadenasso et al., 2003). We hypothesize that assemblages located at ecoregion ecotones have species with more changes from the ancestral character state (higher transition rates), have maintained the ancestral character state for shorter time (lower stasis time), and have more recent transitions to the current character state (lower last transition times) than assemblages from ecoregion cores.

Region cores are more homogeneous environmentally and in terms of selection pressures exerted on organisms, since environmental changes are buffered before they reach cores (Donoghue & Edwards, 2014; Mayle et al., 2004; Mayle & Power, 2008). Populations inhabiting region cores should be large and stable in size over time, as well as occur under environmental conditions similar to the conditions found in the ancestral range (Davies & Buckley, 2011; Pearman et al., 2008; Wiens & Graham, 2005). In contrast, region ecotones are more heterogeneous, both environmentally and in relation to selection pressures exerted on organisms, because environmental challenges are first noticed in ecotones, which leads to changes in vegetation development and in the location, quality and type of habitats, and limiting resources on which individuals depend (De Vivo & Carmignotto, 2004; Donoghue & Edwards, 2014; Eckert et al., 2008; Sexton et al., 2009). Populations inhabiting ecotones should be smaller, be under stronger extinction pressure, and have less stable population size than core populations (Karanth et al., 2006). They also should show shifts from ancestral characters as these shifts may be needed to persist in ecotones (Benton, 2010; Donoghue & Edwards, 2014; Pearman et al., 2008; Sexton et al., 2009).

**FIGURE 1:**
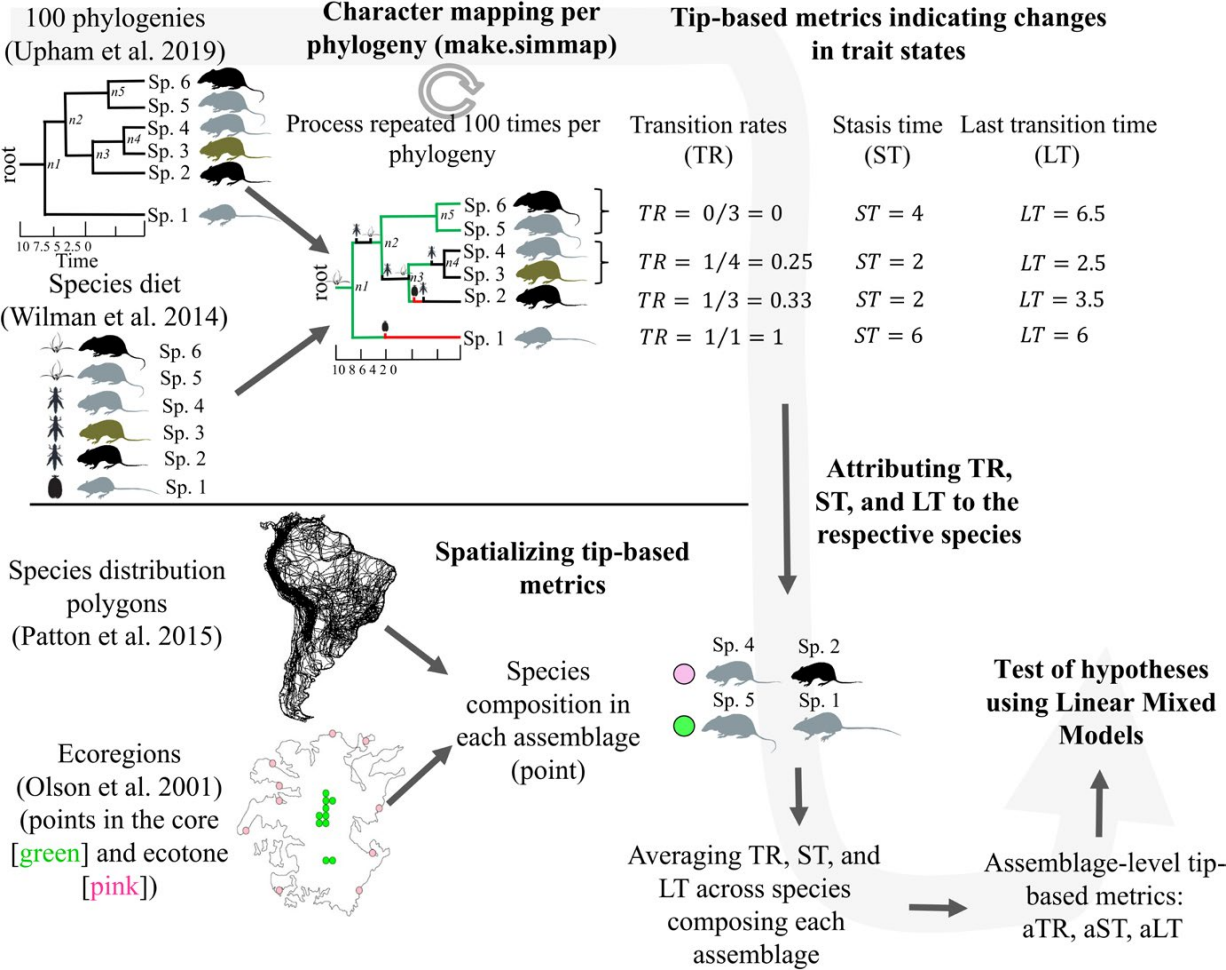
Analytical scheme used to test whether evolution is faster at ecotones, which involved (1) calculating tip‐based ancestral trait state and its change over time and (2) spatializing changes from the ancestral trait state using assemblage‐level metrics (aTR, aST, aLT), and (3) propagating uncertainty across the previous steps (gray arrow in the background). To calculate tip‐based metrics at the species level, we mapped and estimated ancestral states using stochastic mapping of discrete traits via Bayesian inference, which allows calculating the time at which a trait changed along phylogeny nodes. The tip trait state is taken into account when calculating TR (as seen for Sp. 1). Note that transitions not fixed at the nodes are not considered when calculating TR (e.g., the brief transitions between *n1* to *n2* from plant → insect to insect → plant), although such brief transitions do reduce ST and LT. Also note that ST is the maximum time length between two nodes, and LT is the sum of branch lengths with reconstructed traits equal to the tip trait. Values of tip‐based metrics are equal for sister species (Sp. 6 and 5, Sp. 4 and 3) because trait change occurred exactly in the same nodes

Spatial heterogeneity in the rates of species diversification and trait evolution is well known (Benton, 2010; Jetz et al., 2012; Oliveira et al., 2016). But while there are a few metrics to evaluate spatial heterogeneity in rates of diversification (including the tip‐based metrics reviewed in Title & Rabosky, 2018), there is no consensus or metric on how to evaluate spatial heterogeneity in rates of trait evolution. In order to evaluate our hypothesis, we propose three tip‐based metrics for calculating trait transition rates, stasis time, and last transition time (TR, ST, and LT, respectively). All three metrics aim to calculate species‐specific direction and time of character‐state transitions from the phylogeny tips to the root. Transition rates indicate how many times the ancestral character has changed over time. Stasis time indicates the maximum branch length (time interval) over which the current tip‐character was maintained across the whole phylogeny. Finally, last transition time is the sum of branch lengths from the tip to the prior/previous node with a reconstructed character equal to current tip‐character (Figure 1). To calculate the three tip‐based metrics, we mapped and estimated ancestral states using stochastic mapping of discrete traits via Bayesian inference (Bollback, 2006), which allows calculating the time at which a trait changed along phylogeny branches and not just at the nodes. Tip‐based metrics such as TR, ST, and LT can be later summarized across assemblages, allowing assessments of the effect of spatial, environmental, and historical factors on trait evolution rates. Here, we averaged tip‐based metrics across all species occurring in a given assemblage to obtain assemblage‐level TR, ST, and LT – hereafter aTR, aST, and aLT – to then test whether evolution has been faster at ecotones. The test involved a thorough consideration of phylogenetic uncertainty from character reconstruction to hypothesis testing (Figure 1).

We tested our hypothesis of faster trait evolution at ecotones by integrating data on distribution, diet, and phylogeny of sigmodontine rodents. Sigmodontinae is a subfamily within the family Cricetidae (Musser & Carleton, 2005) that arrived in South America before the final closure of the Isthmus of Panama (~10 Ma; Leite et al., 2014; Parada et al., 2021; Steppan & Schenk, 2017). They are a useful group for testing our hypothesis because the species are sensitive to habitat stability at fine scales due to their small body size, short generation time, small geographic range, and narrow microhabitat requirements for feeding, reproducing, and avoiding predation (Patton et al., 2015). One notable aspect about sigmondontine rodents is the uncertain phylogenetic relationships among species, genera, and tribes (although very recently Parada et al. (2021) made important improvements in terms of resolving doubtful relationships between tribes). Different phylogenies show differing but equally plausible topologies (Leite et al., 2014; Machado et al., 2013; Steppan & Schenk, 2017; Weksler, 2006), suggesting that reliance on only one phylogeny may be insufficient to understand the evolution of the group (Range et al., 2015; Upham et al., 2019). Another remarkable aspect about them is that, since their colonization of South America, they experienced a rapid evolutionary radiation that has allowed sigmodontine species to spread into many types of habitats (Patton et al., 2015), without radical changes from their ancestral morphology (Maestri et al., 2017). However, sigmodontine rodents do show an impressive variation in diet (Missagia et al., 2019; Paglia et al., 2012). Many species are specialized to consume a few items from specific habitats, such as herbs and seeds from open habitats or leaves and fruits from forested habitats (Paglia et al., 2012; Pardiñas et al., 2020; Verde Arregoitia & D’Elia, 2021). Thus, diet should evolve as a response to the spatial heterogeneity and temporal instability of ecotones.

## 2 MATERIALS AND METHODS

### 2.1 The unit of analysis

In our analyses, we used the 93 World Wildlife Foundation ecoregions of the Neotropics (Olson et al., 2001) included within the total extent of rodent range maps (−55.98º S to 12.63º N, −86.07º E to −34.79º W). By design, the WWF ecoregions consider regional species pools, represent homogeneous areas in terms of biota and climates, capture major environmental heterogeneity at a global scale, and are objectively classifiable into major habitat types (Olson et al., 2001; Smith et al., 2018). Furthermore, ecoregion ecotones represent meaningful boundaries between biological communities (Smith et al., 2018), a property highly desirable considering our hypothesis. The Neotropical ecoregions embrace a striking diversity of habitats and have changed in position due to climate change over geological time (Costa et al., 2017). Furthermore, such changes were more severe at ecoregion ecotones than at their cores (Mayle et al., 2004; Mayle & Power, 2008).

Spatial analyses used maps in Lambert cylindrical equal‐area projection centered on the center of South America (15ºS, 56ºW). We began our analyses by building an empty raster of cells of ~26 km^2^ (~0.25º cell in a Lat‐Long coordinate system). We used this cell size to ensure sufficient sample size in ecotones and cores. Next, we determined the coordinates of the cell centroid of ecoregions in order to obtain points at several distances from ecoregion ecotones (Figure 1). For each ecoregion, we measured the geographical distance between each point and points at the ecoregion boundary using the *pointDistance* function (“raster” package, Hijmans, 2020). As we were interested in comparing the tip‐based metrics between points at the ecotone and core, we defined ecotone points as the 10 points closest to the ecoregion boundary, whereas we defined core points as the 10 points farthest from the ecoregion boundary (Figure 1). Our total sample size was 1860 points: 930 in cores and 930 in ecotones from 93 different ecoregions.

We obtained the identity of sigmodontine rodents whose ranges overlap the centroid points in the core and ecotone of ecoregions. We used a buffer of ~13.2 km (half of cell size, equivalent to ~0.125‐degree width in a Lat‐Long coordinate system) to obtain the species composition around the points; a width of 13.2 km also avoided the overlap between buffers, which would result in high spatial dependence in rodent composition between neighboring points. After obtaining point‐scale composition, we continued the analyses with species occurring exclusively in the ecotone or core of each ecoregion. We used the range maps of 350 of 384 species listed in Patton et al. (2015) for which we could calculate tip‐based metrics. Maps available in Patton et al. (2015) were formulated based on expert knowledge, and represent the most up‐to‐date source of rodent distribution data. Nomenclature and classification mainly followed accounts in Patton et al. (2015), updated where necessary (see Maestri et al., 2017 and references therein). Range maps are available in Dryad Digital Repository (Maestri et al., 2019, http://dx.doi.org/10.5061/dryad.8vt6s95).

### 2.2 Phylogeny

We used one of the most updated and complete sets of phylogenies of sigmodontine rodents (Upham et al., 2019) for ancestral diet mapping and phylogenetic uncertainty analysis. The phylogenies have dated branches and were built from a supermatrix alignment of 11 genes which were extracted from a more inclusive mammalian supermatrix of 31 genes (Upham et al., 2019). The phylogenies were built using the multigene approach for 279 of 413 extant sigmodontine species; the remaining 134 species were randomly placed at the tips of the phylogeny according to prevailing taxonomy. To consider phylogenetic uncertainty, we used a random sample of 100 phylogenies. An ancestral state was estimated for each node included in the evolutionary history of the 413 species, but we focused our analysis on the 350 species with distribution data that were included in the phylogeny. The mapping of ancestral characters was repeated for the 100 phylogenies in order to assess uncertainty in tip‐based metrics (see below *Ancestral character mapping*).

### 2.3 Diet states

We used the percentage of animal and vegetal items included in the diet of sigmodontine rodents from the Elton Traits v.1 database (Wilman et al., 2014). The database has suitable resolution to characterize rodent diet with high detail. We allocated each rodent species to one of four diet states: (1) insectivores (≥50% of the diet comprised by insects, <50% of the diet comprised by plants and fruits/seeds); (2) plant‐eaters (≥50% plants, <50% of insects and fruits/seeds); (3) fruit and seed‐eaters (≥50% fruits/seeds, <50% of insects and plants); and (4) generalists (several types of food items composing <50% of the diet). We used these percentage cut‐offs because most sigmodontine species are omnivorous (Maestri et al., 2017; Paglia et al., 2012; Patton et al., 2015). Thus, few of them would be included in a non‐omnivore group if we were to use higher percentage cut‐offs. Since diet data were lacking for 33 of the 350 species having distribution and phylogenetic data, we imputed the percentage of consumed items for these species using a random forest algorithm without the phylogeny (Stekhoven & Buehlmann, 2012) so as not to force a relationship between imputed traits and phylogeny (which were later used in ancestral character mapping). Recent work shows that the random forest algorithm can still produce robust imputation estimates even without a phylogeny, especially where there is a low proportion of missing data (as is the case here) (Debastiani et al., 2021).

### 2.4 Ancestral character mapping

We used an algorithm of stochastic mapping of discrete characters via Bayesian inference (Bollback, 2006) to reconstruct the trajectory of diet states across the rodent phylogenies. We used the function *make*.*simmap* of the Phytools package (Revell, 2012), implemented in the R environment. Stochastic mapping based on Bayesian inference allows calculation of the discrete ancestral state (s) of the phylogeny's nodes and the timing of changes along the branches. Stochastic mapping output shows the most probable ancestral state (s) of a node; this output is based on the mean posterior probability of finding a given state and the timing of changes along the phylogeny branches. The mean posterior probability is based on a sample of the posterior probability across a desired number of simulations; here, we used 100 phylogenies and 100 simulations per phylogeny.

Evolutionary processes can produce both symmetric and asymmetric transitions across diet states (Joy et al., 2016), so we first defined whether transitions across diet states are equal (“SYM”, symmetric model) or different (“ARD”, all‐rates‐different model) (Table S1, see Supplementary Methods). As the model with symmetric transition rates had more support than the model with asymmetric rates, we conducted definitive stochastic character mapping using the complete set of 100 phylogenies, with 100 simulations per phylogeny, to more robustly estimate parameters under the symmetric evolutionary model. The output of the stochastic mapping procedure consisted of a set of 10,000 estimates of diet states and length of time that a given diet state persisted per node. This time length is based on the branch length between two nodes with a common diet state. To attribute diet category and estimated time to each node, we built an adjacency matrix with phylogeny tips (species) in the rows, and internal‐node numbers in the columns. Values of 1 were attributed to nodes belonging to the evolutionary history of a species. The first column is the phylogeny root and is completely filled with 1’s, as it belongs to the evolutionary history of all species; the last column is the most recent internal node leading to a tip. These 1’s were then replaced by the reconstructed diet category and time. When more than one state was equally probable at a given node, we used the state present longer at that node.

### 2.5 Tip‐based metrics

The estimated node states were used to calculate three tip‐based metrics of trait evolution. The transition rates (TR) of the species *s* were calculated as:

$$\text{TRs}=\frac{t}{N}$$

where *t* is the number of transitions of trait states detected at the nodes that a species underwent from the phylogeny root to the tip, and *N* is the total number of nodes, counting from the tip to the root. A value equal to 1 indicates that the species presented as many character‐state transitions as possible given its evolutionary history, whereas a value equal to 0 indicates that there were no transitions – the tip and the ancestral character‐state remained the same (Figure 1).

Stasis Time (ST) of the species *s* was calculated as:

$${\text{ST}}_{s}=\max\left\{L_{i},\ldots ,L_{N}\right\}\text{if}\;L_{i}\in A_{i}=a$$

where L is the branch length value from node *i* to *N* that have the trait‐state *A* similar to the tip trait‐state *a*. Stasis time (ST) examines evidence for character retention over time (Figure 1). The metric consists in determining, across the whole phylogeny, the maximum value of branch length between two nodes with mapped trait *A* similar to the tip trait *a*. This can be seen in Figure 1, where species 3 is currently an insectivore, having recently transitioned from a plant‐eater diet. Its lineage had a longer time as an insect‐eater from node 2 to 3 than between any other nodes. Thus, the longer stasis time as an insect‐eater is that one embracing the branches predating the time as a plant‐eater.

Finally, Last Transition Time (LT) of the species *s* was calculated as:

$${\text{LT}}_{s}=\sum_{i=1}^{\text{min}\left\{N,L_{i}\notin A_{i}=a\right\}}L_{i}$$

where branch lengths *L* are summed from node *i* to *N* having a trait A similar to the tip trait *a*. The sum stops when the trait *A* of the node *i* differs from the tip trait *i* = 1. Last transition time indicates when the current tip trait became fixed. The values of LT will exceed ST because the former consists of a sum of more recent branch lengths with trait *a*, whereas the latter is the maximum branch length between two nodes with trait *a*. R code with the tip‐based metrics we develop here can be found in the GitHub of the first author.

### 2.6 Ecoregion‐scale variables

We tested whether tip‐based metrics varied relative to ecoregion ecotone and cores, as well as to other variables characterizing the ecological and biogeographic context of ecoregions (Table 1). First, we superimposed the points on the ecoregion shapefile (Olson et al., 2001) to determine whether points were in ecoregion cores or ecotones, and whether their habitat was either forested or open. The distinction between forested and open habitats reflects broad differences in vegetation structure and in the type of available niches and resources (Vivo & Carmignotto, 2004). Ecoregions belonging to forest, woodland, and mangrove biomes were considered forested habitats, while ecoregions belonging to grassland, shrubland, desert, savanna, inland‐water, and the rock and ice biomes were considered as open habitats.

**TABLE 1:** Ecoregion‐scale variables. All variables were measured at a point scale

Variable	Type	Mean ± SD/factor levels
Position	Factor	Core^b^, ecotone
Habitat	Factor	Forest, open^b^
Position × habitat type interaction	Factor	Core‐forest, core‐open^b^, ecotone‐forest, ecotone‐open
Sum of neighbors’ area^a^	Quantitative	9.03 ± 29.02 square degrees
Number of points overlapping neighbor open‐habitat ecoregions^a^	Quantitative	0.494 ± 0.739
Number of points overlapping neighbor forest‐habitat ecoregions^a^	Quantitative	1.044 ± 1.114
Point location at the Atlantic rainforest region	Factor	1 = within Atlantic Rainforest; 0 = not within Atlantic Rainforest^b^
Point location at the Andean region	Factor	1 = within Andes; 0 = not within Andes^b^

Abbreviation: SD, Standard deviation.

^a^ Standardized to zero mean and unit variance before analysis.

^b^ Factor level represented by the intercept.

We considered the predominant habitat type and number of neighboring ecoregions in our analyses. Neighborhood can be important because an ecotone assemblage can have values of tip‐based metrics that resemble a core assemblage when the ecotone lies between two ecoregions with similar habitat. That ecotone assemblage is then expected to have lower transition rates, higher stasis time, and longer last transition times than an assemblage located in the ecotone between ecoregions with contrasting habitats.

We considered the importance of the Central Andes and Atlantic Forest in predicting aTR, aST, and aLT because sigmodontine diversification and richness have a close relationship with these regions (Maestri & Patterson, 2016; Maestri et al., 2019; Patton et al., 2015). We superimposed ecoregions on the shapefiles of Central Andes (Löwenberg‐Neto, 2015) and the Atlantic Rainforest (Muylaert et al., 2018, available at https://github.com/LEEClab/ATLANTIC‐limits) to distinguish their ecoregions from others (Figure S1, see Supplementary Results). We treated the southernmost portions of the Andean region (mainly southern Argentina and Andean piedmont), as well as extreme northern and southern portions of Atlantic Rainforest as belonging to other regions (e.g., Uruguayan Savanna, Cerrado, Caatinga), not as primary loci of sigmodontine diversification (Maestri et al., 2019).

### 2.7 Statistical analyses

#### 2.7.1 Testing the influence of ecoregion‐scale variables, ecoregion identity, and spatial autocorrelation

We averaged species‐level tip‐based metrics across species of an assemblage to obtain tip‐based metrics at the level of ecological assemblage (hereafter: aTR, aST, aLT) and run hypothesis test (Figure 1).

We estimated the effect of ecoregion‐scale variables on each assemblage‐level tip‐based metric using linear mixed models (LMM, Pinheiro & Bates, 2000). Linear mixed models are a class of models that allow estimating the effect of grouping factors describing the study design (random effect), of spatial autocorrelation (as an error term), and of interesting ecological processes (as a fixed effect, Table 1) when modeling variation in aTR, aST, and aLT. Here ecoregion identity was considered as random effect in LMM analysis as they were part of the sampling design, and differences in shape and convolutedness could mask differences between cores and ecotones.

We identified high spatial autocorrelation (Moran's *I* > 0.5, *p* < .001) for all tip‐based metrics analyzed here. We then looked for spatial autocorrelation in residuals of our LMM models with either aTR, aST, or aLT as response variables, ecoregion‐scale variables as fixed effects (Table 1), and ecoregion identity as a random effect. Spatial autocorrelation was incorporated in the model through an exponential correlation structure with nugget effect (i.e., spatial autocorrelation at very short spatial distances) based on the latitude and longitude values of each point. We used exponential structure because the variograms generally showed a highly stepped decrease in spatial autocorrelation, mainly between very close points; we allowed for a nugget effect to capture such a variation between spatially close points. Comparisons of models with and without nugget effect generally supported the model with nugget effect (Table S2).

To account for phylogenetic uncertainty on tip‐based metrics we ran one LMM analysis per estimate of aTR, aST, and aLT. We accounted for phylogenetic uncertainty using a randomly subsampled set of 2000 of the 10,000 estimated values, due to computational limitations when estimating fixed, random, and spatial parameters for the whole dataset of estimates. Thus, uncertainty on random effect (standard deviation, *σ*), spatial autocorrelation (range, *r* and nugget, *n*), and fixed effect (regression intercept, and regression coefficient of each variable) were represented by the standard deviation calculated across estimates from the 2000 models. The LMM intercept represents the average tip‐based metric when quantitative variables are at their average (i.e., zero in the standardized scale), and factors are at their first level of contrast (Table 1). The regression coefficient of each variable represents the number of standard deviations from the intercept: the larger the coefficient, the stronger the effect of a variable on the response variable (Schielzeth, 2010). We used density plots to represent and infer the effect of ecoregion‐scale variables because these plots can show the most likely average parameter value and effect size, as suggested by most of phylogenies. Boxplots in the margins represent the average, first, and third quartiles of the distribution of parameter estimates across the 2000 models.

#### 2.7.2 The basis of phylogenetic uncertainty

We evaluated whether phylogenetic uncertainty arises from phylogeny structure or stochastic character mapping. To do so we used a randomization procedure, repeated 1000 times. In each step of this procedure, we took 10 estimates of each tip‐based metric produced by simulations within one phylogeny, and 10 estimates of each tip‐based metric produced by one simulation of 10 different phylogenies. We then calculated the standard deviation of pooled estimates, and counted the number of randomizations that the standard deviation was lower within than between phylogenies.

#### 2.7.3 Sensitivity analysis

A strong ecotone effect could be found for assemblages from ecoregions having many small‐ranged species, as these species are more likely to have their distribution centered in the ecoregion core, as well high abundance and occurrence probability at ecoregions’ core (Andrewartha & Birch, 1954; Brown, 1984). Sigmodontine rodents have, in general, small range sizes (min = 0.02 square degrees, 1st quartile = 4.16, median = 18.30, mean = 55.46, 3rd Quartile = 51.40, max = 797.37 square degrees, measured across the 350 species included in our dataset), and many species have their range totally included within the area of a unique ecoregion (Figures S5 and S6). Furthermore, the number of species having a range smaller than ecoregion area varies geographically. While most small‐ranged species occur in the Andes, we observed a considerable number of such species in Atlantic Rainforest, Cerrado, Chaco, and southwest Amazonian regions (Figures S5–S7).

We evaluated whether results would change when analyzing aTR, aST, and aLT of assemblages of small‐ranged species. To avoid area effects when classifying small‐ranged species as those having their range smaller than ecoregion area (Figure S5), we considered as small‐ranged species those having a range size smaller than 4.16 square degrees, the 1st quartile of range‐size values presented above. These models included values of aTR, aST, and aLT across 88 small‐ranged sigmodontine species, distributed in 58 ecotone points of 14 different ecoregions, and in 81 core points of 31 different ecoregions (Figure S6).

#### 2.7.4 Mapping assemblage‐level tip‐based metrics across space

We considered the assemblage‐level tip‐based metrics derived from all 10,000 estimates (100 ancestral character simulations for each of the 100 phylogenies) to build maps showing spatial variation on average and uncertainty (standard deviation) of aTR, aST, and aLT.

#### 2.7.5 Relationship between assemblage‐level tip‐based metrics and species richness

We used a Generalized Least Squares (GLS) regression (Pinheiro & Bates, 2000) to analyze the effect of assemblage richness on aTR, aST, and aLT, because tip‐based metrics can be high for assemblages with many species. We used GLS to account for spatial correlation in the relationship between richness and tip‐based metrics. Correlation structure was exponential with nugget effect — the same we used in LMMs. Spatial and statistical analyses were conducted using packages “raster” (Hijmans, 2019), “sp” (Bivand et al., 2013), and “nlme” (Pinheiro et al., 2020) in R 4.0.2 (R Core Team, 2020).

#### 2.7.6 Relationship between assemblage‐level tip‐based metrics and phylogenetic diversity

We used the same GLS regression just described to test the influence of phylogenetic diversity on aTR, aST, and aLT, because tip‐based metrics can be high for assemblages composed by phylogenetically distinct species. Furthermore, we also tested whether phylogenetic diversity — the sum of the phylogenetic branch lengths connecting species of a community (Swenson, 2014) — varies between ecotones and cores. Ecotone assemblages can consist of species from different neighboring regions, thus resulting in higher phylogenetic diversity and likely higher values of tip‐based metrics in ecotones than cores. Phylogenetic diversity was calculated according to the function written by Swenson (2014).

## 3 RESULTS

Sigmodontine species showed an average of 3.05 ± 0.50 diet transitions, and their evolutionary history had an average of 11.78 ± 2.50 nodes across phylogenies and reconstructions. The average dietary transition rate was $\bar{\mathrm{TR}}=0.26\pm 0.12$, average stasis time was $\bar{\mathrm{ST}}=2.50\pm 0.61$ millions of years, and the average last transition time was $\bar{\mathrm{LT}}=5.76\pm 1.36$ millions of years across species, phylogenies, and reconstructions.

### 3.1 Influence of ecoregion‐scale variables

We found that ecotone assemblages had lower aTR, higher aST and aLT than core assemblages (Table 2). However, the effect of position on aTR, aST, and aLT was generally stronger when it interacted with habitat type (Table 2). Position × habitat type interaction had the largest coefficient in the model of aTR (Table 2), and the second largest coefficient in the model of aST. The isolated effect of position had the fourth largest coefficient in the model of aLT (Table 2).

**TABLE 2:** Average parameter value ± standard deviation representing phylogenetic uncertainty on estimates of fixed effects, random effect, and spatial correlation structure across 2000 linear mixed models

Effect/variable	Transition rates	Stasis time	Last transition times
	Average estimate ± standard deviation		
Fixed effect			
Intercept	0.263 ± 0.121	2.502 ± 0.605	5.760 ± 1.356
Position (ecotone)	−0.001 ± 0.010	0.005 ± 0.063	0.059 ± 0.199
Habitat type	0.000 ± 0.031	−0.061 ± 0.133	0.104 ± 0.620
Position × habitat interaction	0.004 ± 0.012	0.045 ± 0.080	−0.039 ± 0.272
Sum of neighbor's area	0.001 ± 0.004	0.025 ± 0.025	0.024 ± 0.093
Point overlap with forest‐habitat ecoregions	−0.001 ± 0.003	−0.016 ± 0.019	−0.024 ± 0.081
Point overlap with open‐habitat ecoregions	0.000 ± 0.005	−0.025 ± 0.021	0.040 ± 0.130
Atlantic rainforest ecoregions	−0.001 ± 0.030	0.011 ± 0.136	−0.576 ± 0.721
Andean ecoregions	0.002 ± 0.034	0.040 ± 0.171	0.157 ± 0.695
Random effect			
Standard deviation (*σ*)	0.029 ± 0.021	0.145 ± 0.127	0.740 ± 0.505
Residual	0.075 ± 0.022	0.453 ± 0.156	2.052 ± 0.398
Spatial correlation structure			
Range (*r*)^a^	108.7 ± 107.52	139.83 ± 128.01	90.314 ± 103.14
Nugget (*n*)	0.186 ± 0.010	0.206 ± 0.106	0.172 ± 0.093

Note

Fixed effects are represented in standard deviations from the intercept for each assemblage‐level tip‐based metric (columns).

^a^ Scale of kilometers.

**FIGURE 2:**
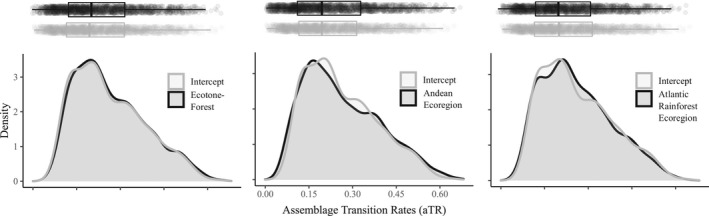
Density plots of the intercept (expected mean) of assemblage transition rates aTR, and regression coefficient (deviation from the mean) of the most important variables. In each plot, the intercept is represented by the gray line and the regression coefficient is represented by the black line. Estimates were extracted from Linear Mixed Models that consider ecoregion‐scale variables as fixed effects, ecoregion ID as random effect, and exponential correlation structure with nugget effect to accommodate spatial autocorrelation. Intercept and regression coefficients were extracted from each one of the 2000 models. Boxplot in the upper margin shows average and 1st and 3rd quartiles of the distribution of aTR

Although there was substantial phylogenetic uncertainty on parameter estimates, as observed by the range of values along the *x*‐axis of the density plot, we found that assemblages at the ecotone of forested ecoregions had higher aTR than the expected aTR average (Figure 2). Other influential variables for aTR were Andean and Atlantic Rainforest ecoregions (Table 2). Assemblages at Andean ecoregions had subtly higher aTR than the expected aTR average, whereas assemblages at Atlantic Rainforest ecoregions had subtly lower aTR than the expected aTR average (Table 2, Figure 2). Density plots of least important coefficients — the ones with small regression coefficients (Table 2) and generally tight overlap of intercept and coefficient estimates — for each tip‐based metric can be found in the Supplementary Results (Figures S2–S4).

**FIGURE 3:**
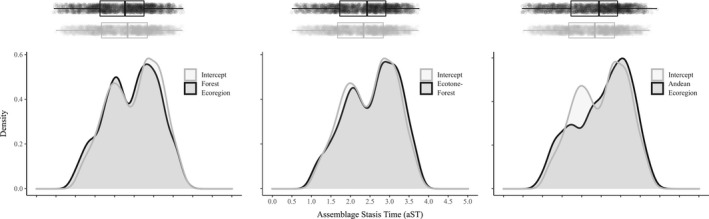
Density plots of the intercept (expected mean) of assemblage stasis time aST (millions of years), and regression coefficient (deviation from the mean) of the most important variables. In each plot, the intercept is represented by the gray line and the regression coefficient is represented by the black line. Estimates were extracted from Linear Mixed Models that consider ecoregion‐scale variables as fixed effects, ecoregion ID as random effect, and exponential correlation structure with nugget effect to accommodate spatial autocorrelation. Intercept and regression coefficients were extracted from each one of the 2000 models. Boxplot in the upper margin shows average and 1st and 3rd quartiles of the distribution of aST

Habitat type, position × habitat type interaction, and location at Andean ecoregions were the variables causing the largest deviation in aST from the aST average (Table 2). Although there was substantial phylogenetic uncertainty, with different groups of phylogenies leading to two different peaks of aST estimates, we found that assemblages at forest ecoregions had lower aST than the expected aST average, whereas assemblages at the ecotone of forested ecoregions generally had higher aST than the expected aST average (Figure 3). Assemblages in Andean ecoregions had higher aST than the expected aST average (Figure 3).

**FIGURE 4:**
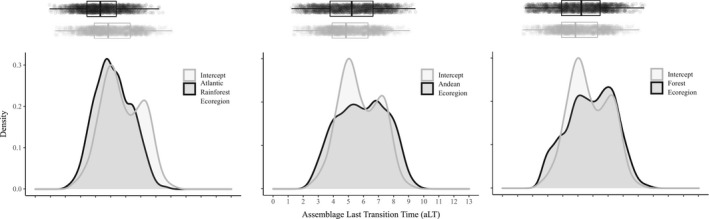
Density plots of the intercept (expected mean) of assemblage last transition time aLT (millions of years), and regression coefficient (deviation from the mean) of the most important variables. In each plot, the intercept is represented by the gray line and the regression coefficient is represented by the black line. Estimates were extracted from Linear Mixed Models that consider ecoregion‐scale variables as fixed effects, ecoregion ID as random effect, and exponential correlation structure with nugget effect to accommodate spatial autocorrelation. Intercept and regression coefficients were extracted from each one of the 2000 models. Boxplot in the upper margin shows average and 1st and 3rd quartiles of the distribution of aLT

Location in the Atlantic Rainforest, location in Andean ecoregions, and habitat type were the variables causing the largest deviation in aLT from aLT average (Table 2). Although phylogenetic uncertainty again affected parameter estimates, we found that assemblages in the Atlantic Rainforest generally had shorter last transition time than the expected aLT average (Figure 4). In contrast, assemblages in Andean ecoregions had longer aLT than the expected aLT average (Figure 4). Finally, assemblages in forest ecoregions had longer aLT than the expected aLT average (Figure 4).

### 3.2 The basis of phylogenetic uncertainty

Standard deviation across estimates of assemblage‐level tip‐based metrics was generally lower within than between different phylogenies. More specifically, the standard deviation of aTR was lower within than between phylogenies in 89% of the randomizations. The standard deviation was also lower in 89% of the randomizations for aST estimates, and 67% for aLT estimates.

### 3.3 Sensitivity analysis

In general, the model with exponential spatial autocorrelation and without the nugget effect was the most supported by the data of small‐ranged species, although there were larger uncertainties in finding the best model and estimating spatial autocorrelation parameters when compared to the complete dataset (Table S3). We found position × habitat type interaction among the most important variables explaining aTR and aST (but not aLT) of assemblages of small‐ranged species, although uncertainty was even larger than the uncertainty observed in previous analysis (Tables S3 and S4, Figures S8–S10). Habitat type, location in the Andes, and position × habitat type interaction were the variables causing the largest deviation in aTR from the expected aTR average (Table S4, Figure S8). Location in Atlantic Rainforest and Andes, and position × habitat interaction were the variables causing the largest deviation in aST from aST average (Table S4, Figure S9). Location in Atlantic Rainforest and Andes, and point overlap with forest‐habitat neighbors were the variables causing the largest deviation in aLT from aLT average (Table S4, Figure S10).

### 3.4 Mapping assemblage‐level tip‐based metrics

**FIGURE 5:**
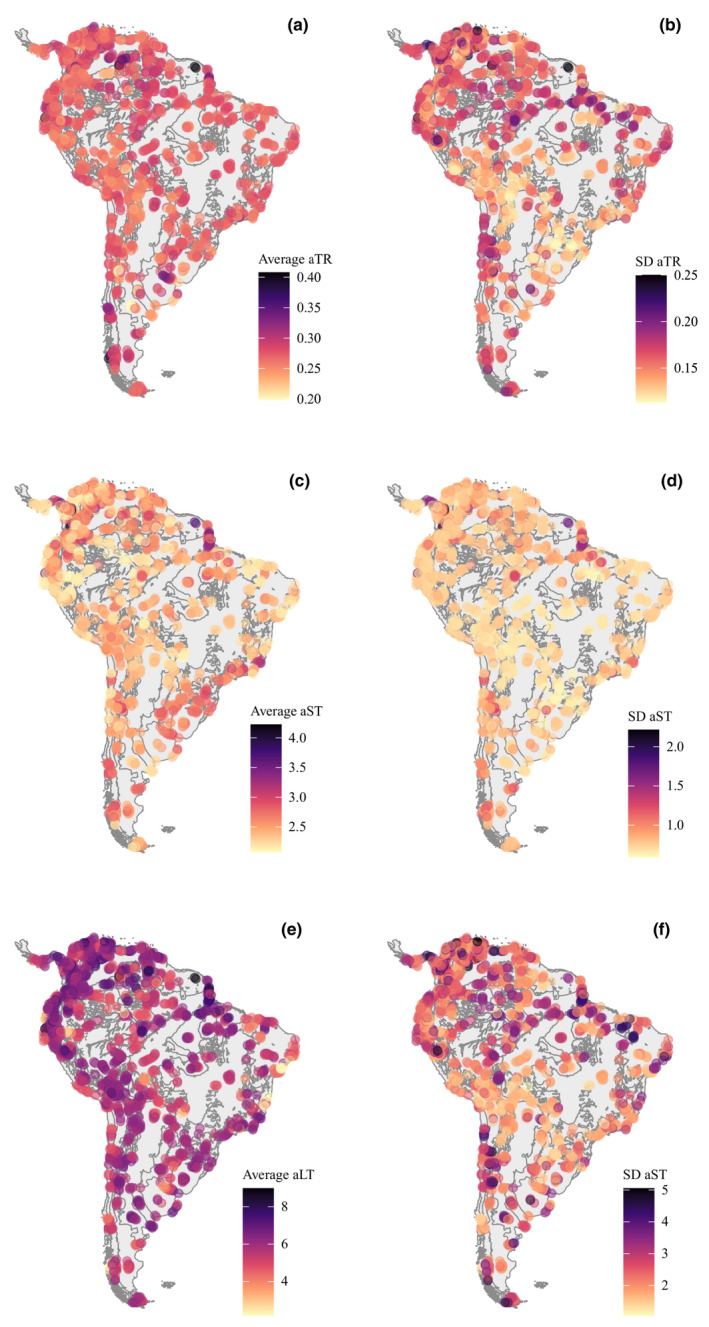
Mapped assemblage‐level transition rates (aTR), stasis time (aST), and last transition time (aLT) of sigmodontine rodent assemblages at points in ecoregion cores and ecotones. Tip‐based metrics in the left maps (a, c, e) were obtained by averaging metrics across 10,000 estimates (100 phylogenies, 100 simulations per phylogeny). Phylogenetic uncertainty on estimates of the tip‐based metrics, represented in the right maps (b, d, f), were calculated through the standard deviation of the metrics across 10,000 estimates. Maps in Lambert Equal‐Area Projection

Spatial variation on assemblage‐level transition rates (aTR), averaged across 10,000 estimates, revealed high aTR in rodent assemblages from central Amazonia, and northwestern and southern South America, and low aTR along the eastern portion of Andes (Figure 5a). Phylogenetic uncertainty in aTR was high in central Amazonia, northeastern South America, and central Andes (Figure 5b). We found high assemblage‐level stasis time (aST) in northwestern, northern and southern South America, and along the Atlantic Rainforest, whereas low aST in northeastern and center South America, and eastern portions of the Andes (Figure 5c). Phylogenetic uncertainty in aST was particularly high where we found the most extreme values of aST (except in the Atlantic Rainforest) (Figure 5d). Finally, we found high aLT across most South America, with low aLT found in assemblages from northeastern South America (particularly in the north of the Atlantic Rainforest) (Figure 5e). Phylogenetic uncertainty in aLT was high in assemblages from central Amazonia, southern and northern South America (Figure 5f).

### 3.5 Random effects and spatial autocorrelation

Just a minor variation in all assemblage‐level tip‐based metrics was explained by ecoregion identity (random effect *σ*, Table 2). Spatial autocorrelation in turn was strong and present even at very small spatial distances (as shown by parameters *r* and *n*, Table 2).

### 3.6 Correlation between tip‐based metrics

Linear correlation between assemblage‐level tip‐based metrics was always lower than 0.5 (Table S5 in Supplementary results).

### 3.7 Relationship of tip‐based metrics with species richness and phylogenetic diversity

We observed no to weak effect of assemblage richness and phylogenetic diversity on aTR, aST, and aLT (Figures S11 and S12 in Supplementary results). We did not find differences in phylogenetic diversity between ecotones and cores (Figure S13).

## 4 DISCUSSION

We found that environmentally heterogeneous ecotones influenced the speed of trait evolution, affecting both the rate and time of diet transitions in sigmodontine rodents. We make such an inference on the rate and timing of trait evolution by developing three tip‐based metrics. These metrics were largely independent from richness and phylogenetic diversity and, at least for the group under study, might provide new information about species‐level trait evolution that can be explored at macroevolutionary (by summarizing values of species within, e.g., genera) and macroecological scales (as done here by mapping trait evolution across the space). We found that the diet of ecotone species changed little from the ancestral diet when compared to core species. Furthermore, an interaction between position and habitat type — which had a stronger effect on the rate and timing of diet transitions than the isolated effect of either position or habitat type — indicated that a broader environmental context dictates the rates and time of diet evolution. Spatial variation on assemblage‐level tip‐based metrics revealed regions with slow‐ and fast‐evolving species. Finally, phylogenetic uncertainty can influence the estimates of rates and time of trait evolution, as well as the inference about the effect of variables on such estimates.

### 4.1 Ecotone effect on assemblage‐level tip‐based metrics

We assumed that current ecoregions have existed more or less continuously over the history of sigmodontine rodents in the Neotropics, and their ecotones have changed more in position and habitat than cores (Donoghue & Edwards, 2014; Mayle et al., 2004; Mayle & Power, 2008; further discussed below in *Study limitations*). Therefore, we hypothesized higher diet transition rates, lower stasis time, and shorter transition times in ecotone than core assemblages. Actually, we found that, relative to core species, ecotone species presented (1) fewer transitions in diet over their evolutionary history, (2) longer periods between diet transitions, and (3) longer retention of the current diet.

The existence of patches of favorable habitat can prevent evolution at ecotone zones. Patches of favorable, high‐quality habitat can be ephemeral and sparsely distributed along ecotones, but can sustain large population sizes with individuals presenting little or no shifts in ecological, morphological, and behavioral characters over time (Eckert et al., 2008; Sexton et al., 2009). These processes result in few transitions from ancestral diet, longer stasis time, and retention of the current diet because the retention of an optimal feeding strategy enables species persistence in ecotone zones. This strategy could be a generalist diet that generally evolves as an option to explore resources from different habitats (Price et al., 2012). Also, patches of a favorable habitat along ecotones can provide the stability needed to maintain the current diet since long time ago, perhaps since late Miocene or early Pliocene when major sigmodontine tribes diversified (Leite et al., 2014; Parada et al., 2021; Steppan & Schenk, 2017) and within‐clade morphological disparity increased (Maestri et al., 2017).

We found a stasis time of around 2.5 ma for both core and ecotone species. It is a long time period under little to no trait evolution relative to the ~10 ma of sigmodontine presence in the Neotropics. Although we do not know the exact geological period in which diet stasis occurred, cooling periods such as the one embracing late Miocene and early Pliocene (Amidon et al., 2017) may well have facilitated diet retention over large time periods. Cooling periods through the Cenozoic are related to speciation slowdowns across major tetrapod clades, likely due to the influence of temperature on the environment's carrying capacity (Condamine et al., 2019).

### 4.2 Habitat effect on assemblage‐level tip‐based metrics

We found that the type of ecoregion habitat — and therefore a broader environmental context — has a large influence on the time of diet evolution. Species from the ecotone of forest ecoregions had higher aTR, and higher aST and subtly lower aLT than the core of forest ecoregions, the core of non‐forest ecoregions, and the ecotone of non‐forest ecoregions. Stability of forest regions can explain prolonged retention of the current diet by species from forest ecoregions. Available evidence show that cores were more stable over time than ecotones, at least for forested regions (Costa et al., 2017; Mayle et al., 2004; Mayle & Power, 2008). This stability can be traced back to the Eocene, which had forests that resemble forests today in terms of vegetation structure and taxonomic composition (Burnham & Johnson, 2004). However, the ecotones of forest ecoregions considerably changed of location and repeatedly expanded over savannas and grasslands over time (Behling & Pillar, 2007; Costa et al., 2017). Rodent feeding strategies may have changed due to ecotone dynamics, then resulting in diet transitions to track variation in available resources. In the same line of evidence, we find that trait evolution is faster at the ecotone of forested ecoregions. The extensive dynamics of forests over non‐forested regions could therefore have demanded more adaptations of sigmodontine rodents to persist in the more forested landscapes of South America.

### 4.3 Phylogenetic uncertainty

We acknowledged phylogenetic uncertainty throughout our analyses, which result in high overlap of parameter estimates (Figures 2, 3, 4, S8–S10). Upham et al. (2019) reported that building their rodent phylogeny was especially challenging due to missing genetic data and topological uncertainty producing polytomies. These uncertainties were further propagated across our estimates of tip‐based metrics. Thus, our inference of traits was based on the collective evidence provided by the phylogenies of Upham et al. (2019).

Results suggest two major implications of phylogenetic uncertainty. The first is the error possible when estimating the average value of the tip‐based metrics. For example, consider the density plot in the middle of Figure 3 where we have two peaks of stasis time. If you choose calculating tip‐based metrics using only one phylogeny it is very likely that you would have an estimate of either ~2 or ~3 ma, but would err by at least ~1 ma. The second implication is the error we can make when estimating the effect size of variables. Again, considering the density plot in the middle of Figure 3, at ~2 ma of stasis time the assemblages from Andean ecoregions had lower stasis time than assemblages located outside this region. However, no difference between these regions can be found at ~3 ma. By propagating uncertainty in our estimates, we infer that the more likely value of average stasis time is ~2.3 ma (inset gray boxplot, Figure 3), and the more likely effect of Andean ecoregions is a positive deviation from average aST (inset black boxplot, Figure 3). Therefore, it is highly desirable that, when available, we use a set of phylogenies rather than only one to test hypothesis about the evolution of ecological traits (Range et al., 2015).

### 4.4 Spatial variation in assemblage‐level tip‐based metrics

Regions in the northern Andes (particularly those near to the Isthmus of Panama), Amazon Basin, and portions of the Atlantic Rainforest have enjoyed environmental stability since the Eocene (Burnham & Johnson, 2004; Costa et al., 2017), and Patagonia and Andean regions suffered few cumulative changes in climate since the Last Glacial Maximum (Sandel et al., 2011). These regions generally present slow‐evolving species (Maestri et al., 2019), high levels of endemism and diversity accumulation over time (Dynnerius & Jansson, 2000; Sandel et al., 2011), and had assemblages of species with low transition rates, high stasis time, and long times since the last diet transition. In contrast, more diet transitions and shorter stasis time and last transition time were found for assemblages disposed along the South American diagonal of open vegetation, a climatically instable region (Costa et al., 2017) that generally present fast‐evolving species (Maestri et al., 2019). These findings highlight that environmental stability favors retention of an ancestral diet.

### 4.5 Study limitations

Environmental variation unrelated to the distance from ecotones could alternatively explain the lower transition rates and higher stasis time and last transition times we find for ecotone species. For instance, ecotones of many ecoregions may be formed by other environmental factors (e.g., soil moisture and type) rather than climate, which might make them temporally more stable than the ecotones formed by climate (Cantidio & Souza, 2019). In addition, patches of stable habitat currently found at ecotones could rather be at the core of ecoregions in the past. Thus, shifts in ancestral area could alternatively influence trait evolution. Combining the tip‐based metrics we developed here with approaches that incorporate shifts in ancestral area (Maestri & Duarte, 2020) can help to show whether trait evolution is produced by shifts in distribution.

We used diet because it varies across sigmodontine species (Paglia et al., 2012) and habitat types (de Vivo & Carmignotto, 2004), and is available for virtually all species (Wilman et al., 2014). In addition, each diet type is subjected to a particular set of selection pressures and presents different probabilities of transition and speeds of evolution (Maestri et al., 2017; Price et al., 2012). Although other important traits like life‐mode could produce different results, we believe our results are robust to trait choice as diet and life‐mode were shown to produce similar macroevolutionary patterns of morphological disparity in sigmodontine rodents (Maestri et al., 2017).

Results were mostly robust when considering small‐ranged species, whose rates and time of diet transition respond to the position × habitat type interaction — similarly to overall results. The location of species assemblages in Andean and Atlantic Rainforest ecoregions has a large influence on its tip‐based metrics. This result is largely expected as most of their small‐ranged species both speciated and subsequently evolved within these regions (D’Elía & Pardiñas, 2015).

## 5 CONCLUSION

Despite considerable phylogenetic uncertainty in the data, we found an influence of ecotone on the rates and timing of diet transitions for sigmodontine rodents. This result is especially noteworthy as there may be only subtle differences in the rates of transition and time of diet evolution between ecotone and core species, owing to the existence of patches of favorable habitat in ecotone zones. The spatial analysis of diet evolution shed light on the evolutionary pathways that sigmodontine rodents tracked to achieve such an impressive diversity, and expand and survive into the large range of habitats in which they occur today. Our approach provides a formal link between macroecology and macroevolution, and can be incorporated in more sophisticated approaches integrating reconstruction of ancestral areas and ecological traits.

## CONFLICT OF INTEREST

We declare that there are no competing interests in relation to this study.

## AUTHOR CONTRIBUTIONS

**André Luís Luza:** Conceptualization (equal); Data curation (equal); Formal analysis (equal); Investigation (equal); Methodology (equal); Software (equal); Visualization (equal); Writing – original draft (equal); Writing – review & editing (equal). **Renan Maestri:** Conceptualization (equal); Formal analysis (equal); Investigation (equal); Methodology (equal); Supervision (equal); Writing – review & editing (equal). **Vanderlei Júlio Debastiani:** Formal analysis (equal); Methodology (equal); Software (equal); Writing – original draft (equal); Writing – review & editing (equal). **Bruce D. Patterson:** Conceptualization (equal); Methodology (equal); Supervision (equal); Writing – review & editing (equal). **Sandra Maria Hartz:** Conceptualization (equal); Funding acquisition (equal); Investigation (equal); Methodology (equal); Resources (equal); Supervision (equal); Writing – original draft (equal); Writing – review & editing (equal). **Leandro D. S. Duarte:** Conceptualization (equal); Formal analysis (equal); Funding acquisition (equal); Investigation (equal); Methodology (equal); Resources (equal); Writing – original draft (equal); Writing – review & editing (equal).

## Supporting information

Appendix S1

## Data Availability

All data we used are already available in online repositories. Range maps are available in the Dryad Digital Repository (http://dx.doi.org/10.5061/dryad.8vt6s95). Phylogenies were published in 2019 by N. Upham and collaborators in PLOS Biology (https://doi.org/10.1371/journal.pbio.3000494). A shapefile with ecoregions is available at https://www.worldwildlife.org/publications/terrestrial‐ecoregions‐of‐the‐world. A shapefile with Central Andes boundaries was published by Löwemberg‐Neto in 2015, and it is available at http://dx.doi.org/10.11646/zootaxa.3985.4.9. A shapefile with Atlantic Rainforest boundaries was published by Muylaert and collaborators in 2018, and it is available at https://github.com/LEEClab/ATLANTIC‐limits. Mammal diet data were published by Wilman and collaborators in 2014 and are available at https://doi.org/10.1890/13‐1917.1. Finally, the R codes used to calculate the three new tip‐based metrics are available on GitHub (https://github.com/andreluza/EvolRates), and codes are data are available on Zenodo (https://doi.org/10.5281/zenodo.5708927).

## References

[ece38476-bib-0001] Amidon, W. H. , Fisher, G. B. , Burbank, D. W. , Ciccioli, P. L. , Alonso, R. N. , Gorin, A. L. , Silverhart, P. H. , Kylander‐Clark, A. R. C. , & Christoffersen, M. S. (2017). Mio‐Pliocene aridity in the south‐central Andes associated with Southern Hemisphere cold periods. Proceedings of the National Academy of Sciences, 114, 6474–6479. 10.1073/pnas.1700327114 PMC5488932 28607045

[ece38476-bib-0002] Andrewartha, H. G. , & Birch, L. C. (1954). The distribution and abundance of animals (pp. 782). University of Chicago Press.

[ece38476-bib-0003] Antonelli, A. , Zizka, A. , Carvalho, F. A. , Scharn, R. , Bacon, C. B. , Silvestro, D. , & Condamine, F. L. (2018). Amazonia is the primary source of Neotropical biodiversity. Proceedings of the National Academy of Sciences, 115, 6034–6039. 10.1073/pnas.1713819115 PMC6003360 29760058

[ece38476-bib-0004] Behling, H. , & Pillar, V. D. (2007). Late Quaternary vegetation, biodiversity and fire dynamics on the southern Brazilian highland and their implication for conservation and management of modern Araucaria forest and grassland ecosystems. Philosophical Transactions of the Royal Society B: Biological Sciences, 362, 243–251. 10.1098/rstb.2006.1984 PMC2311428 17255033

[ece38476-bib-0005] Benton, M. J. (2010). The origins of modern biodiversity on land. Philosophical Transactions of the Royal Society B: Biological Sciences, 365, 3667–3679. 10.1098/rstb.2010.0269 PMC2982001 20980315

[ece38476-bib-0006] Bivand, R. S. , Pebesma, E. , & Gomez‐Rubio, V. (2013). Applied spatial data analysis with R (2nd ed.). Springer. https://asdar‐book.org/

[ece38476-bib-0007] Bollback, J. P. (2006). SIMMAP: Stochastic character mapping of discrete traits on Phylogenies. BMC Bioinformatics, 7, 88. 10.1186/1471-2105-7-88 16504105 PMC1403802

[ece38476-bib-0008] Brown, J. H. (1984). On the relationship between abundance and distribution of species. American Naturalist, 124, 255–279. 10.1086/284267

[ece38476-bib-0009] Burnham, R. J. , & Johnson, K. R. (2004). South American palaeobotany and the origins of neotropical rainforests. Philosophical Transactions of the Royal Society of London. Series B: Biological Sciences, 359, 1595–1610. 10.1098/rstb.2004.1531 15519975 PMC1693437

[ece38476-bib-0010] Cadenasso, M. L. , Pickett, S. T. A. , Weathers, K. C. , & Jones, C. G. (2003). A Framework for a Theory of Ecological Boundaries. BioScience, 53, 750–758.

[ece38476-bib-0011] Cantalapiedra, J. L. , FitzJohn, R. G. , Kuhn, T. S. , Fernández, M. H. , DeMiguel, D. , Azanza, B. , Morales, J. , & Mooers, A. Ø. (2014). Dietary innovations spurred the diversification of ruminants during the Caenozoic. Proceedings of the Royal Society B: Biological Sciences, 281, 20132746. 10.1098/rspb.2013.2746 PMC3871323 24352949

[ece38476-bib-0012] Cantidio, L. S. , & Souza, A. F. (2019). Aridity, soil and biome stability influence plant ecoregions in the Atlantic Forest, a biodiversity hotspot in South America. Ecography, 42, 1887–1898.

[ece38476-bib-0013] Condamine, F. L. , Rolland, J. , & Morlon, H. (2019). Assessing the causes of diversification slowdowns: temperature‐dependent and diversity‐dependent models receive equivalent support. Ecology Letters, 22, 1900–1912. 10.1111/ele.13382 31486279

[ece38476-bib-0014] Costa, G. C. , Hampe, A. , Ledru, M.‐P. , Martinez, P. A. , Mazzochini, G. G. , Shepard, D. B. , Werneck, F. P. , Moritz, C. , Carnaval, A. C. , & Fortin, M.‐J. (2017). Biome stability in South America over the last 30 kyr: Inferences from long‐term vegetation dynamics and habitat modelling. Global Ecology and Biogeography, 27, 285–297. 10.1111/geb.12694

[ece38476-bib-0015] D’Elía, G. , & Pardiñas, U. F. J. (2015). Subfamily Sigmodontinae Wagner, 1843. In J. L. Patton , U. F. J. Pardiñas , & G. D’Elía (Eds.), Mammals of South America, vol. 2: Rodents (pp. 63–70). Univ. of Chicago Press.

[ece38476-bib-0016] Davies, T. J. , & Buckley, L. B. (2011). Phylogenetic diversity as a window into the evolutionary and biogeographic histories of present‐day richness gradients for mammals. Philosophical Transactions of the Royal Society B: Biological Sciences, 366, 2414–2425. 10.1098/rstb.2011.0058 PMC3130431 21768156

[ece38476-bib-0017] de Vivo, M. , & Carmignotto, A. P. (2004). Holocene vegetation change and the mammal faunas of South America and Africa. Journal of Biogeography, 31, 943–957. 10.1111/j.1365-2699.2004.01068.x

[ece38476-bib-0018] Debastiani, V. J. , Bastazini, V. A. G. , & Pillar, V. D. (2021). Using phylogenetic information to impute missing functional trait values in ecological databases. Ecological Informatics, 63, 101315. 10.1016/j.ecoinf.2021.101315

[ece38476-bib-0019] Donoghue, M. J. , & Edwards, E. J. (2014). Biome shifts and niche evolution in plants. Annual Review of Ecology Evolution and Systematics, 45, 547–572. 10.1146/annurev-ecolsys-120213-091905

[ece38476-bib-0020] Dynnerius, M. , & Jansson, R. (2000). Evolutionary consequences of changes in species’ geographical distributions driven by Milankovitch climate oscillations. Proceedings of the National Academy of Sciences, 97, 9115–9120. 10.1073/pnas.97.16.9115 PMC16831 10922067

[ece38476-bib-0021] Eckert, C. G. , Samis, K. E. , & Lougheed, S. C. (2008). Genetic variation across species’ geographical ranges: the central–marginal hypothesis and beyond. Molecular Ecology, 17, 1170–1188. 10.1111/j.1365-294X.2007.03659.x 18302683

[ece38476-bib-0022] Gingerich, P. D. (2009). Rates of Evolution. Annual Review of Ecology Evolution and Systematics, 40, 657–675. 10.1146/annurev.ecolsys.39.110707.173457

[ece38476-bib-0023] Goldberg, E. E. , Roy, K. , Lande, R. , & Jablonski, D. (2005). Diversity, endemism, and age distributions in macroevolutionary sources and sinks. American Naturalist, 165, 623–633. 10.1086/430012 15937743

[ece38476-bib-0024] Hijmans, R. J. (2019). geosphere: Spherical Trigonometry. R package version 1.5‐10. https://CRAN.R‐project.org/package=geosphere

[ece38476-bib-0025] Hijmans, R. J. (2020). Raster: Geographic Data Analysis and Modeling. R package version 3.1‐5. https://CRAN.R‐project.org/package=raster

[ece38476-bib-0026] Jetz, W. , Thomas, G. H. , Joy, J. B. , Hartmann, K. , & Mooers, A. O. (2012). The global diversity of birds in space and time. Nature, 491, 444–448. 10.1038/nature11631 23123857

[ece38476-bib-0027] Joy, J. B. , Liang, R. H. , McCloskey, M. , Nguyen, T. , & Poon, A. F. Y. (2016). Ancestral Reconstruction. PLOS Computational Biology, 12, e1004763. 10.1371/journal.pcbi.1004763 27404731 PMC4942178

[ece38476-bib-0028] Karanth, K. K. , Nichols, J. D. , Sauer, J. R. , & Hines, J. E. (2006). Comparative dynamics of avian communities across edges and interiors of North American ecoregions. Journal of Biogeography, 33, 674–682. 10.1111/j.1365-2699.2005.01392.x

[ece38476-bib-0029] Leite, R. N. , Kolokotronis, S.‐O. , Almeida, F. C. , Werneck, F. P. , Rogers, D. S. , & Weksler, M. (2014). In the wake of invasion: tracing the historical biogeography of the South American cricetid radiation (Rodentia, Sigmodontinae). PLoS One, 9, e100687. 10.1371/journal.pone.0100687 24963664 PMC4071052

[ece38476-bib-0030] Löwenberg‐Neto, P. (2015). Andean region: a shapefile of Morrone’s (2015) biogeographical regionalisation. Zootaxa, 3985, 600. 26250168 10.11646/zootaxa.3985.4.9

[ece38476-bib-0031] Machado, L. F. , Leite, Y. L. , Christoff, A. U. , & Giugliano, L. G. (2013). Phylogeny and biogeography of tetralophodont rodents of the tribe Oryzomyini (Cricetidae: Sigmodontinae). Zoologica Scripta, 43, 119–130. 10.1111/zsc.12041

[ece38476-bib-0032] Maestri, R. , & Duarte, L. (2020). Evoregions: Mapping shifts in phylogenetic turnover across biogeographic regions. Methods in Ecology and Evolution, 11, 1652–1662. 10.1111/2041-210X.13492

[ece38476-bib-0033] Maestri, R. , Monteiro, L. R. , Fornel, R. , Upham, N. S. , Patterson, B. D. , & Freitas, T. R. O. (2017). The ecology of a continental evolutionary radiation: Is the radiation of sigmodontine rodents adaptive? Evolution, 71–3, 610–632. 10.1111/evo.13155 28025827

[ece38476-bib-0034] Maestri, R. , & Patterson, B. D. (2016). Patterns of species richness and turnover for the south American rodent fauna. PLoS One, 11, e0151895. 10.1371/journal.pone.0151895 26999278 PMC4801412

[ece38476-bib-0035] Maestri, R. , Upham, N. S. , & Patterson, B. D. (2019). Tracing the diversification history of a Neogene rodent invasion into South America. Ecography, 42, 683–695. 10.1111/ecog.04102

[ece38476-bib-0036] Mayle, F. E. , Beerling, D. J. , Gosling, W. D. , & Bush, M. B. (2004). Responses of Amazonian ecosystems to climatic and atmospheric carbon dioxide changes since the last glacial maximum. Philosophical Transactions of the Royal Society of London. Series B: Biological Sciences, 359, 499–514. 10.1098/rstb.2003.1434 15212099 PMC1693334

[ece38476-bib-0037] Mayle, F. E. , & Power, M. J. (2008). Impact of a drier Early–Mid‐Holocene climate upon Amazonian forests. Philosophical Transactions of the Royal Society B: Biological Sciences, 363, 1829–1838. 10.1098/rstb.2007.0019 PMC2374889 18267912

[ece38476-bib-0038] McGill, B. J. , Chase, J. M. , Hortal, J. , Overcast, I. , Rominger, A. J. , Rosindell, J. , Borges, P. A. V. , Emerson, B. C. , Etienne, R. S. , Hickerson, M. J. , Mahler, D. L. , Massol, F. , McGaughran, A. , Neves, P. , Parent, C. , Patiño, J. , Ruffley, M. , Wagner, C. E. , & Gillespie, R. (2019). Unifying macroecology and macroevolution to answer fundamental questions about biodiversity. Global Ecology and Biogeography, 28, 1925–1936. 10.1111/geb.13020

[ece38476-bib-0039] Missagia, R. V. , Patterson, B. D. , & Perini, F. A. (2019). Stable isotope signatures and the trophic diversification of akodontine rodents. Evolutionary Ecology, 33, 855–872. 10.1007/s10682-019-10009-0

[ece38476-bib-0040] Musser, G. G. , & Carleton, M. D. (2005). Superfamily Muroidea. In D. E. Wilson , & D. M. Reeder (Eds.), Mammal species of the world: A taxonomic and geographic reference (pp. 894–1531). The Johns Hopkins University Press.

[ece38476-bib-0041] Muylaert, R. L. , Vancine, M. H. , Bernardo, R. , Oshima, J. E. F. , Sobral‐Souza, T. , Tonetti, V. R. , Niebuhr, B. B. , & Ribeiro, M. C. (2018). Uma nota sobre os limites territoriais da Mata Atlântica. Oecologia Australis, 22, 302–311. 10.4257/oeco.2018.2203.09

[ece38476-bib-0042] Oliveira, B. , Machac, A. , Costa, G. C. , Brooks, T. M. , Davidson, A. D. , Rondinini, C. , & Graham, C. H. (2016). Species and functional diversity accumulate differently in mammals. Global Ecology and Biogeography, 25, 1119–1130. 10.1111/geb.12471

[ece38476-bib-0043] Olson, D. M. , Dinerstein, E. , Wikramanayake, E. D. , Burgess, N. D. , Powell, G. V. N. , Underwood, E. C. , D'amico, J. A. , Itoua, I. , Strand, H. E. , Morrison, J. C. , Loucks, C. J. , Allnutt, T. F. , Ricketts, T. H. , Kura, Y. , Lamoreux, J. F. , Wettengel, W. W. , Hedao, P. , & Kassem, K. R. (2001). Terrestrial ecoregions of the world: A new map of life on Earth. BioScience, 51, 933–938.

[ece38476-bib-0044] Paglia, A. P. , Fonseca, G. A. B. D. , Rylands, A. B. , Herrmann, G. , Aguiar, L. M. S. , Chiarello, A. G. , & Leite, Y. L. R. (2012). Annotated checklist of Brazilian mammals (2 nd ed., pp. 1–76). Conservation International.

[ece38476-bib-0045] Parada, A. , Hanson, J. , & D’Elía, G. (2021). Ultraconserved Elements Improve the Resolution of Difficult Nodes within the Rapid Radiation of Neotropical Sigmodontine Rodents (Cricetidae: Sigmodontinae). Systematic Biology, 70, 1090–1100. 10.1093/sysbio/syab023 33787920

[ece38476-bib-0046] Pardiñas, U. F. J. , Cañón, C. , Galliari, C. A. , Brito, J. , Hoverud, N. B. , Lessa, G. , & Oliveira, J. A. (2020). Gross stomach morphology in akodontine rodents (Cricetidae: Sigmodontinae: Akodontini): a reappraisal of its significance in a phylogenetic context. Journal of Mammalogy, 101, 835–857. 10.1093/jmammal/gyaa023

[ece38476-bib-0047] Patton, J. L. , Pardiñas, U. F. J. , & D’Elía, G. (Eds.) (2015). Mammals of South America, volume 2: Rodents (pp. 1336). University of Chicago Press.

[ece38476-bib-0048] Pearman, P. B. , Guisan, A. , Broennimann, O. , & Randin, C. F. (2008). Niche dynamics in space and time. Trends in Ecology & Evolution, 23, 149–158. 18289716 10.1016/j.tree.2007.11.005

[ece38476-bib-0049] Pinheiro, J. C. , & Bates, D. M. (2000). Mixed‐effect models in S and S‐plus (pp. 528). Springer.

[ece38476-bib-0050] Pinheiro, J. , Bates, D. , DebRoy, S. , & Sarkar, D. , & R Core Team (2020). nlme: Linear and Nonlinear Mixed Effects Models. R package version 3.1‐148. https://CRAN.R‐project.org/package=nlme

[ece38476-bib-0051] Price, S. A. , Hopkins, S. S. B. , Smith, K. K. , & Roth, V. L. (2012). Tempo of trophic evolution and its impact on mammalian diversification. Proceedings of the National Academy of Sciences, 109, 7008–7012. 10.1073/pnas.1117133109 PMC3345017 22509033

[ece38476-bib-0052] R Core Team (2020). R: A language and environment for statistical computing. R Foundation for Statistical Computing. https://www.R‐project.org/

[ece38476-bib-0053] Range, T. F. , Colwell, R. K. , Graves, G. R. , Fuciková, K. , Rahbek, C. , & Diniz‐Filho, J. A. (2015). Phylogenetic uncertainty revisited: Implications for ecological analyses. Evolution, 69, 1301–1312. 10.1111/evo.12644 25800868

[ece38476-bib-0054] Revell, L. J. (2012). Phytools: an R package for phylogenetic comparative biology (and other things). Methods in Ecology and Evolution, 3, 217–223. 10.1111/j.2041-210X.2011.00169.x

[ece38476-bib-0055] Sandel, B. , Arge, L. , Dalsgaard, B. , Davies, R. G. , Gaston, K. J. , Sutherland, W. J. , & Svenning, J.‐C. (2011). The influence of late quaternary climate‐change velocity on species endemism. Science, 334, 660–664. 10.1126/science.1210173 21979937

[ece38476-bib-0056] Schielzeth, H. (2010). Simple means to improve the interpretability of regression coefficients. Methods in Ecology and Evolution, 1, 103–113. 10.1111/j.2041-210X.2010.00012.x

[ece38476-bib-0057] Sexton, J. P. , McIntyre, P. J. , Angert, A. L. , & Rice, K. J. (2009). Evolution and Ecology of Species Range Limits. Annual Review of Ecology Evolution and Systematics, 40, 415–436. 10.1146/annurev.ecolsys.110308.120317

[ece38476-bib-0058] Smith, J. R. , Letten, A. D. , Ke, P.‐J. , Anderson, C. B. , Hendershot, J. N. , Dhami, M. K. , Dlott, G. A. , Grainger, T. N. , Howard, M. E. , Morrison, B. M. L. , Routh, D. , San Juan, P. A. , Mooney, H. A. , Mordecai, E. A. , Crowther, T. W. , & Daily, G. C. (2018). A global test of ecoregions. Nature Ecology & Evolution, 2, 1889–1896. 10.1038/s41559-018-0709-x 30397301

[ece38476-bib-0059] Stekhoven, D. J. , & Buehlmann, P. (2012). Package MissForest ‐ nonparametric missing value imputation for mixed‐type data. Bioinformatics, 28, 112–118. 10.1093/bioinformatics/btr597 22039212

[ece38476-bib-0060] Steppan, S. J. , & Schenk, J. J. (2017). Muroid rodent phylogenetics: 900‐species tree reveals increasing diversification rates. PLoS One, 12, e0183070. 10.1371/journal.pone.0183070 28813483 PMC5559066

[ece38476-bib-0061] Swenson, N. (2014). Functional and phylogenetic ecology in R (pp. 212). Springer.

[ece38476-bib-0062] Title, P. O. , & Rabosky, D. L. (2018). Tip rates, phylogenies and diversification: What are we estimating, and how good are the estimates? Methods in Ecology and Evolution, 10, 821–834.

[ece38476-bib-0063] Upham, N. S. , Esselstyn, J. A. , & Jetz, W. (2019). Inferring the mammal tree: Species‐level sets of phylogenies for questions in ecology, evolution, and conservation. PLoS Biology, 17, e3000494. 10.1371/journal.pbio.3000494 31800571 PMC6892540

[ece38476-bib-0064] Verde Arregoitia, L. D. , & D’Elia, G. (2021). Classifying rodent diets for comparative research. Mammal Rev, 51, 51–65. 10.1111/mam.12214

[ece38476-bib-0065] Weksler, M. (2006). Phylogenetic relationships of Oryzomine rodents (Muroidea: Sigmodontinae): separate and combined analyses of morphological and molecular data. Bulletin of the American Museum of Natural History, 296(1), 149.

[ece38476-bib-0066] Wiens, J. J. , & Donoghue, M. J. (2004). Historical biogeography, ecology and species richness. Trends in Ecology & Evolution, 19, 639–644. 10.1016/j.tree.2004.09.011 16701326

[ece38476-bib-0067] Wiens, J. J. , & Graham, C. H. (2005). Niche conservatism: integrating evolution, ecology, and conservation biology. Annual Review of Ecology Evolution and Systematics, 36, 519–539. 10.1146/annurev.ecolsys.36.102803.095431

[ece38476-bib-0068] Wilman, H. , Belmaker, J. , Simpson, J. , de la Rosa, C. , Rivadeneira, M. M. , & Jetz, W. (2014). EltonTraits 1.0: Species‐level foraging attributes of the world's birds and mammals. Ecology, 95(7), 2027. 10.1890/13-1917.1

