# Supplementary material for: Is evolution faster at ecotones? A test using rates and tempo of diet transitions in Neotropical Sigmodontinae (Rodentia, Cricetidae)
Source: Ecol Evol. 2021 Dec 16;11(24):18676–90. doi: 10.1002/ece3.8476 (PMC8717272; doi:10.1002/ece3.8476)
Supplement: Supplementary file 1 — Appendix S1 [file ECE3-11-18676-s001.pdf]

Online Supporting information

This file contains:

Supplementary methods

Supplementary results

Supplementary results considering small-ranged species

Supplementary methods

Choosing between symmetric and asymmetric transitions across diet states

Evolutionary processes can produce symmetric or asymmetric transitions across diet states. Thus, we first defined whether transitions across diet states are equal ("SYM", symmetric model) or different ("ARD", all-rates-different model – hereafter 'asymmetric'). To test that, we used the first 10 of the 100 phylogenies of Upham et al. (2019). The transitions of diet states (matrix **Q**) were estimated through Bayesian inference based on 50 simulations of a Monte-Carlo Markov Chain per phylogeny. The value of each phylogeny root was estimated by numerically solving pi***Q**=0, which was then used as a prior for the root value. We also obtained the AIC value of each simulation of both SYMM and ARD models, in order to define the evolutionary model that best characterizes the transitions across diet states. We used a difference in AIC (ΔAIC) larger than two to declare that one model is more supported than another.

The pairwise comparison of AIC values between the two models across a set of 500 estimates showed greater support for symmetric over asymmetric transitions across states. The symmetric model was better supported in 364 of 500 estimates, whereas asymmetric model received more support in 136 of 500 estimates. Average and standard deviation of AIC values over 500 estimates was 726.17 ± 93.18 and 821.06 ± 45.96 for symmetric and asymmetric models, respectively. The transition rates were higher between fruit and seed-eaters with generalists and between fruit and seed-eaters with plant-eaters (Table S1). In general, species from Akodontini tribe were estimated to have an insect-eating ancestor, whereas Phyllotini species were estimated to have a plant-eating ancestor. Thomasomyini were estimated to have a fruit and seed-eating

ancestor, and Oryzomyini were estimated to have a fruit and seed-eater / generalist ancestor (See *Diet reconstructions mapped onto phylogenies* below).

Table S1: Transition matrix, **Q**, showing the frequency of changes of species diet between different diet states estimated by the symmetric model. Averages and standard deviations were calculated across 500 estimates (50 simulations and 10 phylogenetic trees).

	Fruit-eaters	Generalists	Insect-eaters	Plant-eaters
Fruit and seed-eaters	-1.246 ± 1.046			
Generalists	0.383 ± 0.783	-1.471 ± 1.320		
Insect-eaters	0.258 ±0.672	0.305 ± 0.794	-0.610 ± 0.972	
Plant-eaters	0.604 ± 0.800	0.782 ± 1.159	0.047 ± 0.028	-1.434 ± 1.514

Diet reconstructions mapped onto phylogenies

Diet reconstructions mapped onto phylogenies were based on symmetric transitions between states. Preliminary test using 10 of 1,000 phylogenies showed the model including symmetric transition between diets had more support than the asymmetric model. The state of each node was estimated using stochastic mapping of discrete traits via Bayesian inference, and the respective proportion of times a character state has more support (presented as chart plots) was estimated through 50 simulations.

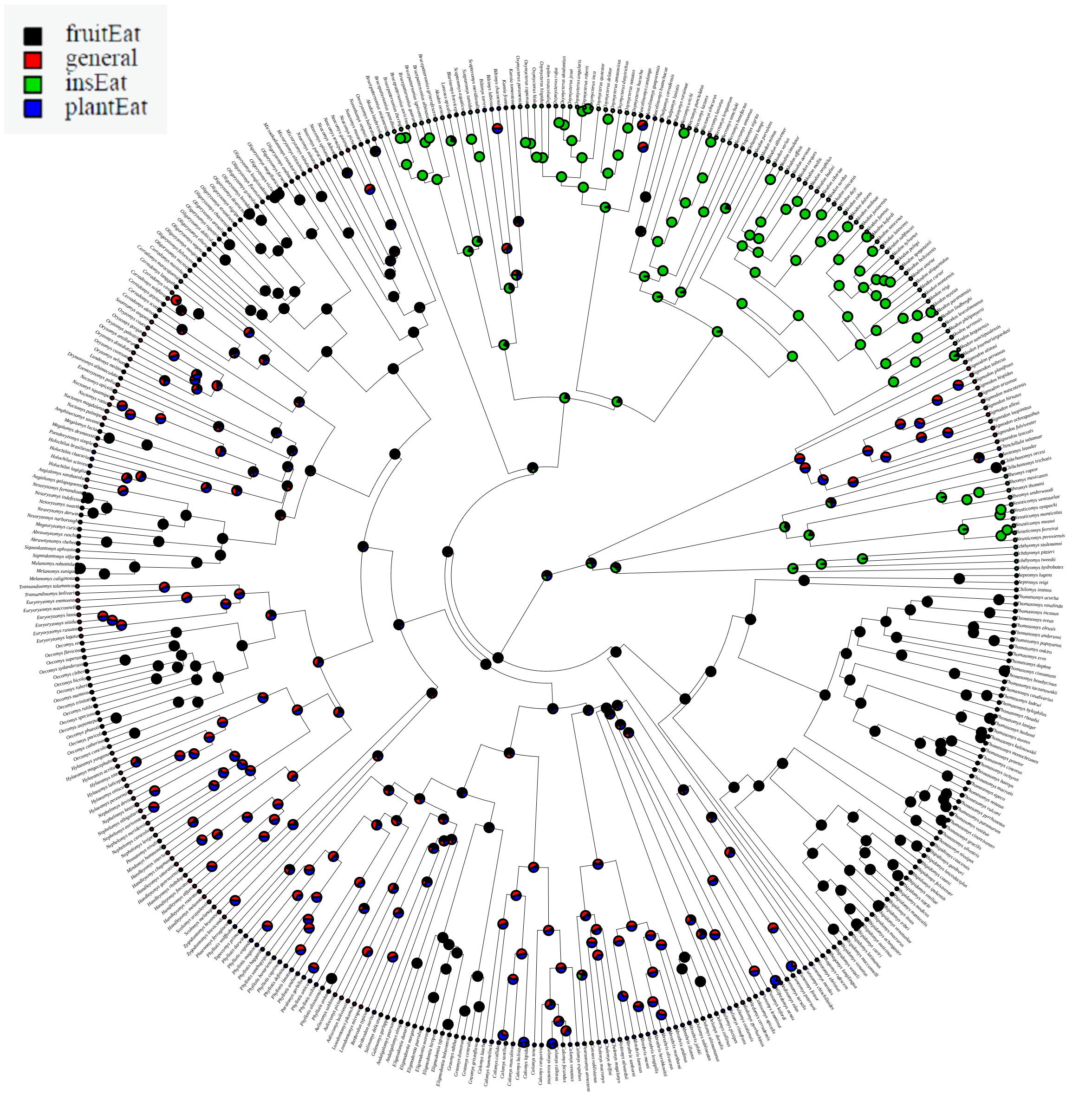
fruitEat
general
insEat
plantEat

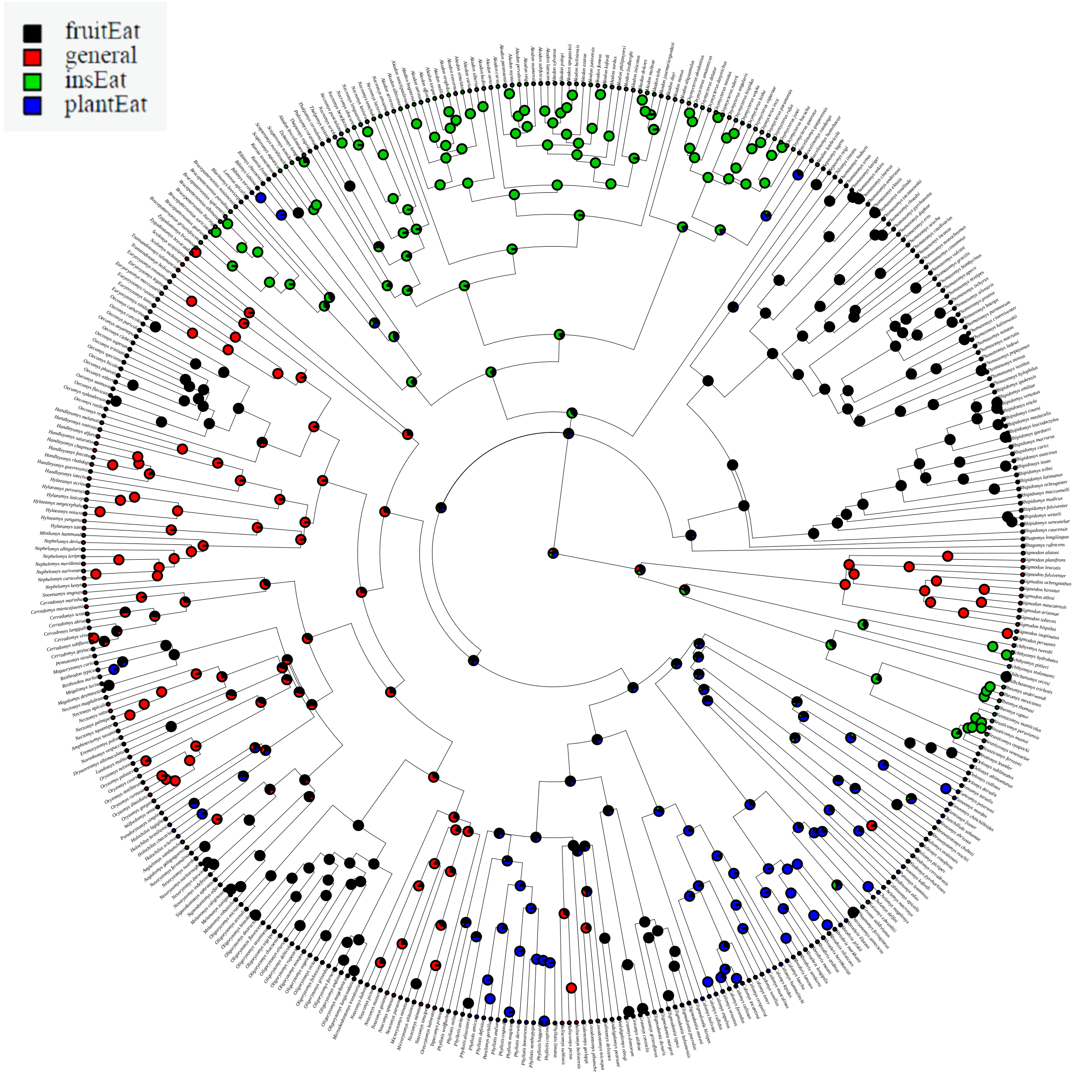
fruitEat
general
insEat
plantEat

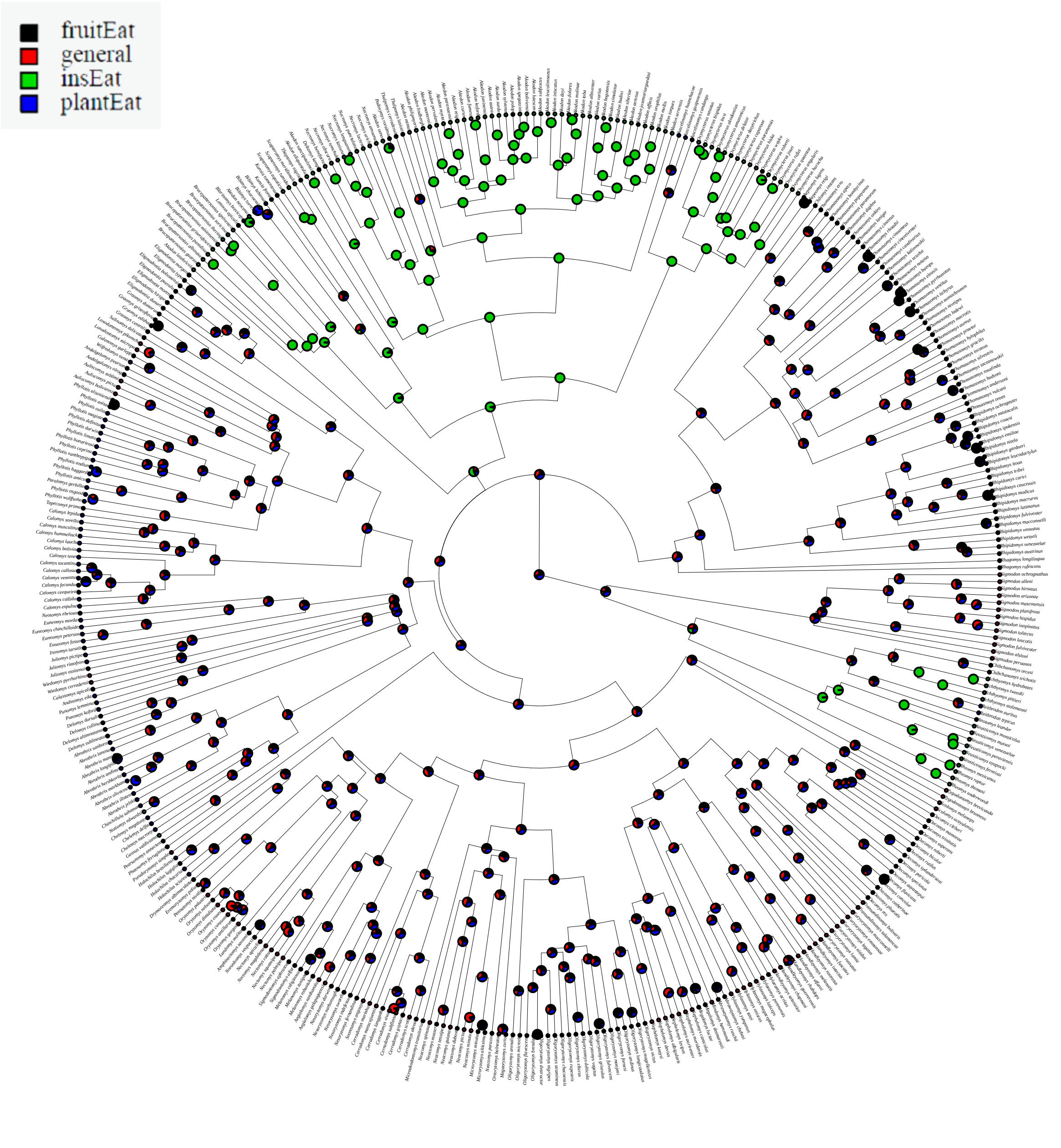
fruitEat
general
insEat
plantEat

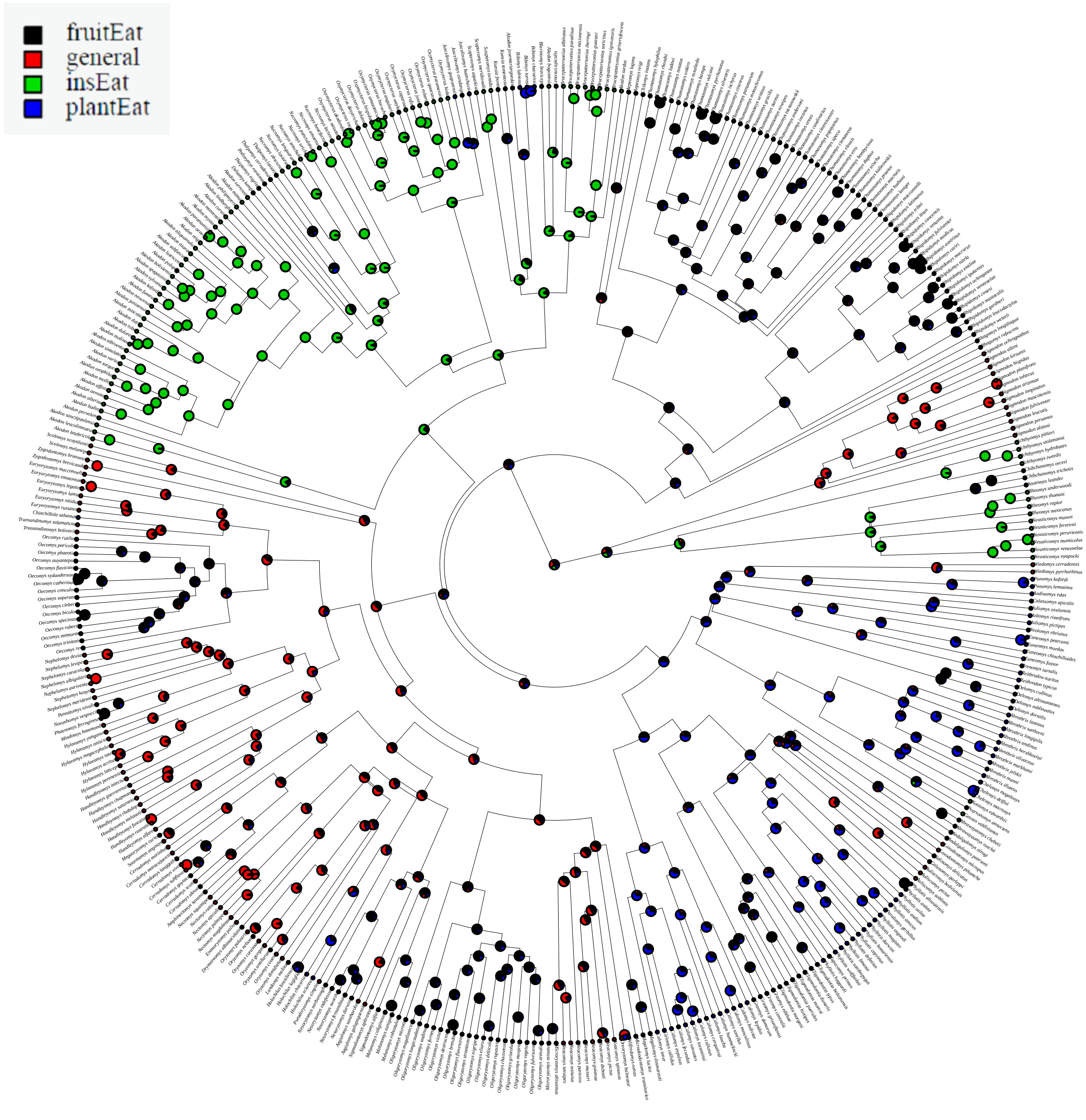
fruitEat
general
insEat
plantEat

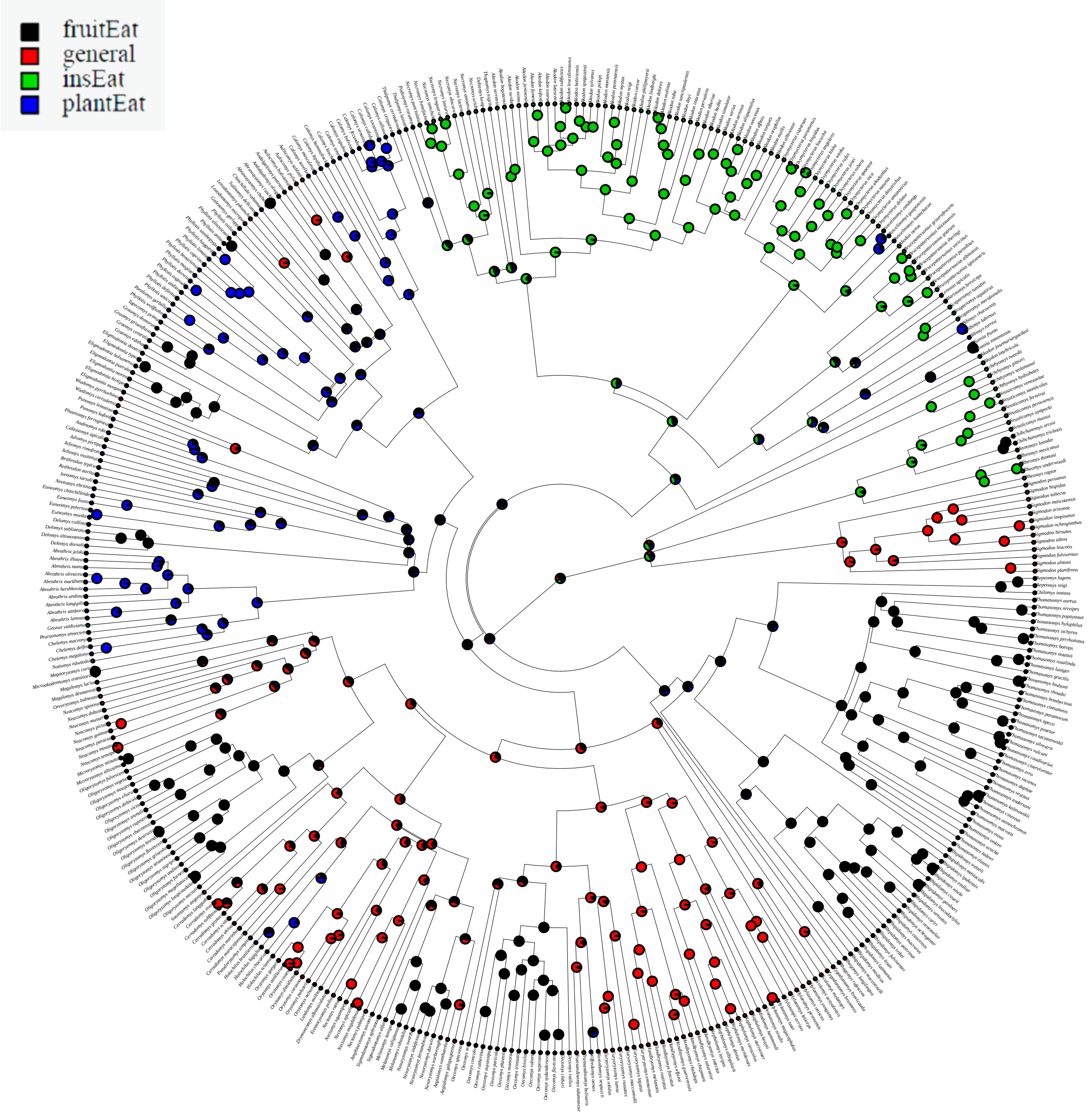
fruitEat
general
insEat
plantEat

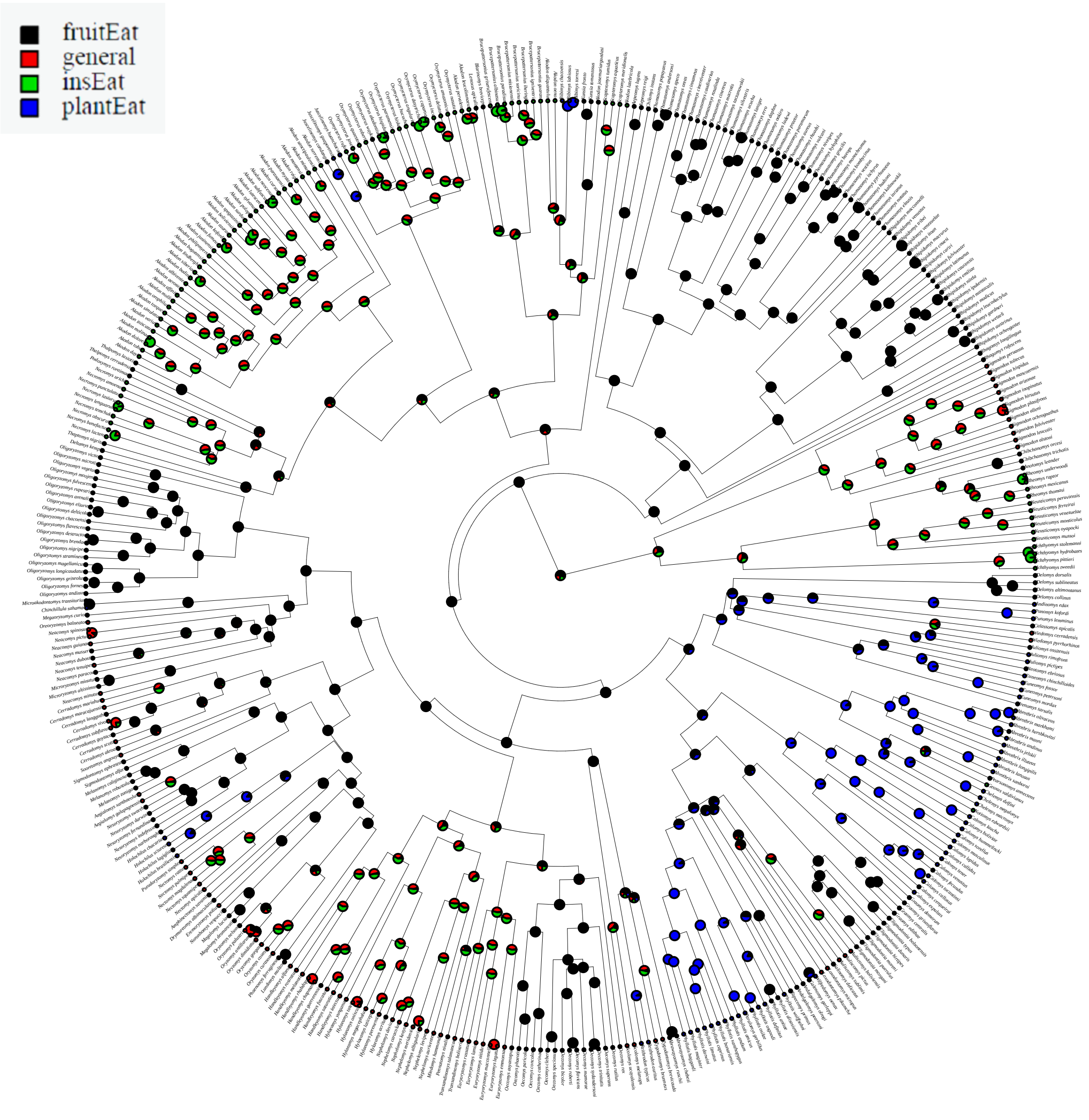
fruitEat
general
insEat
plantEat

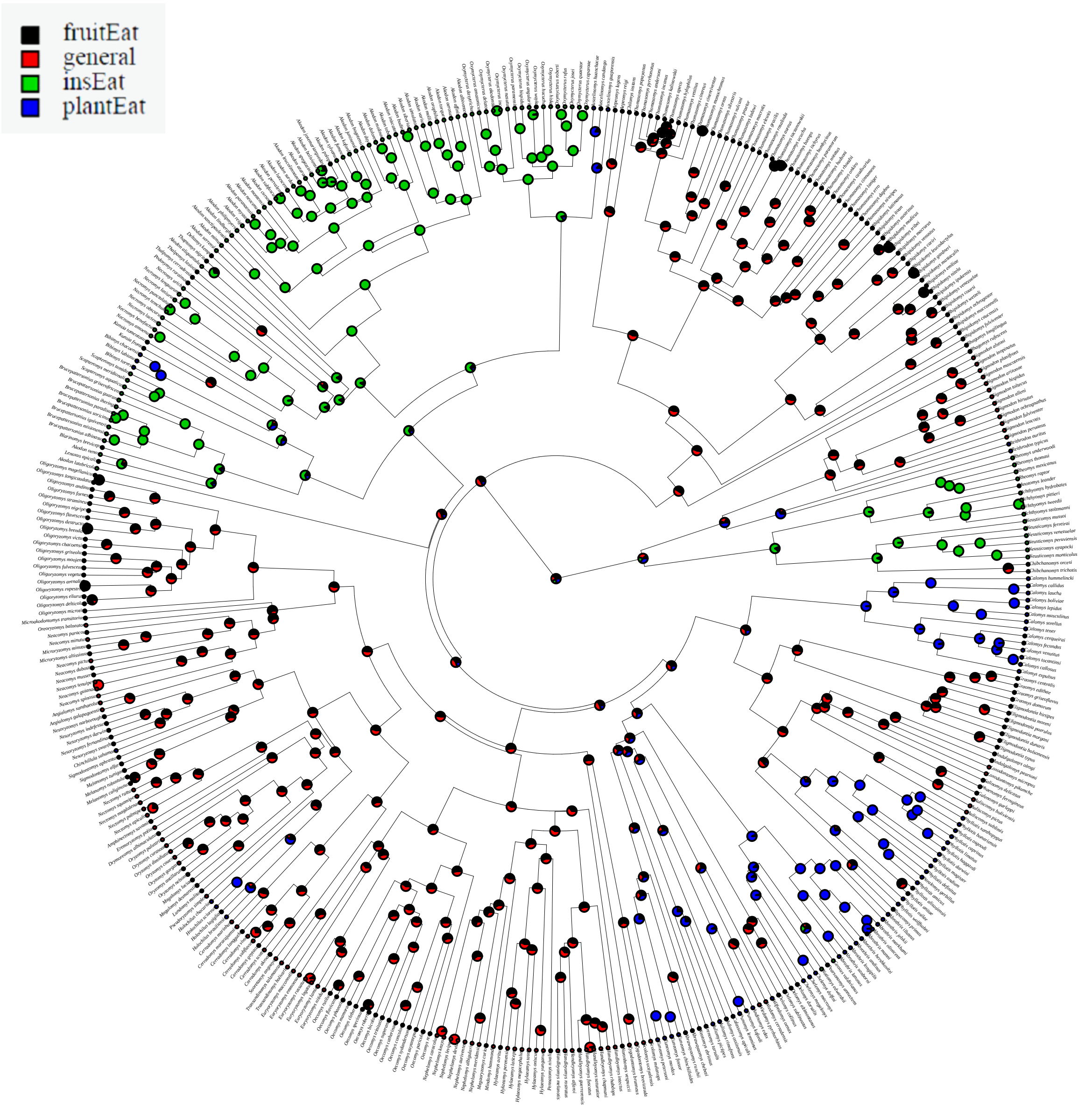

fruitEat
general
insEat
plantEat

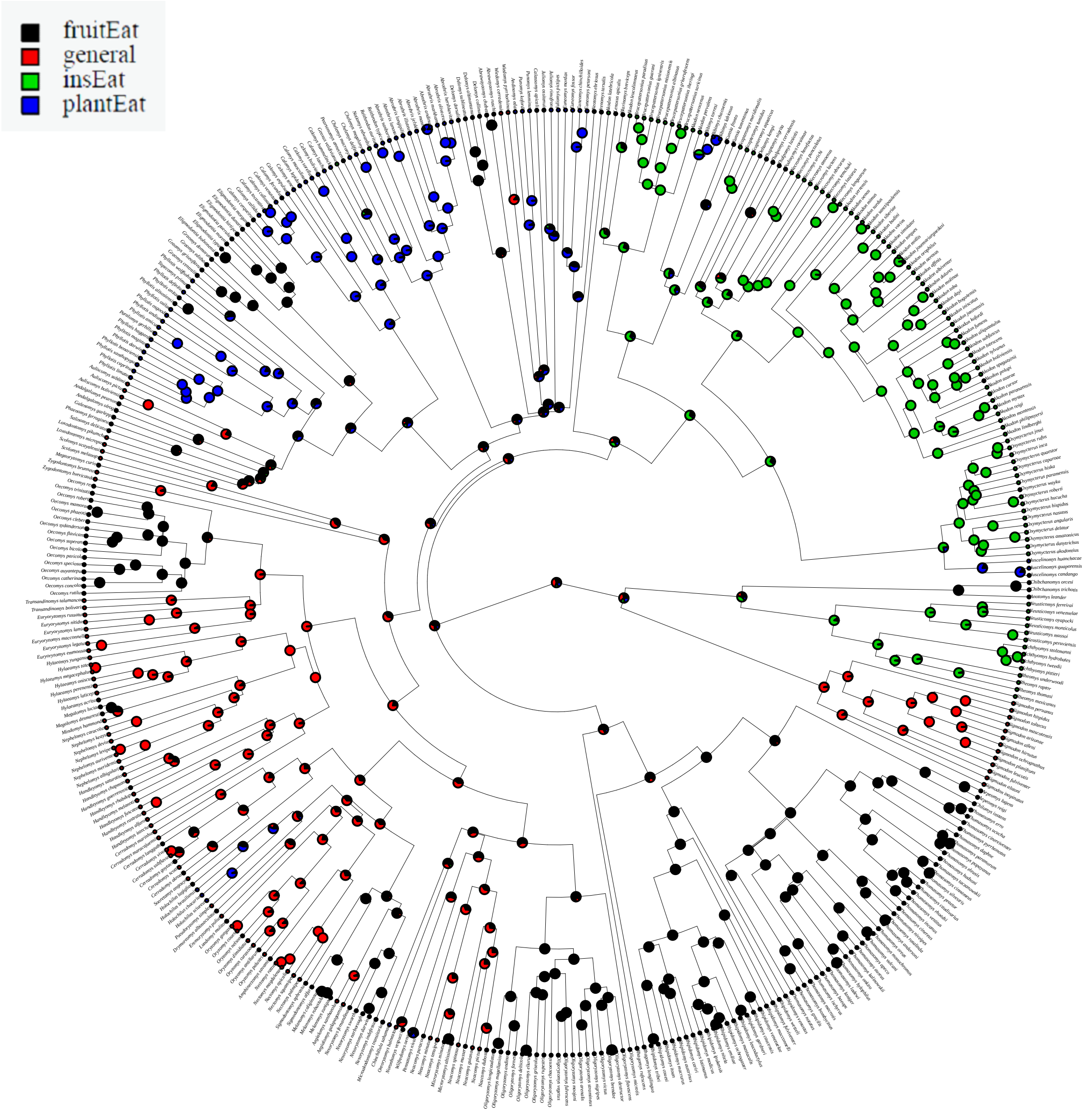
fruitEat
general
insEat
plantEat

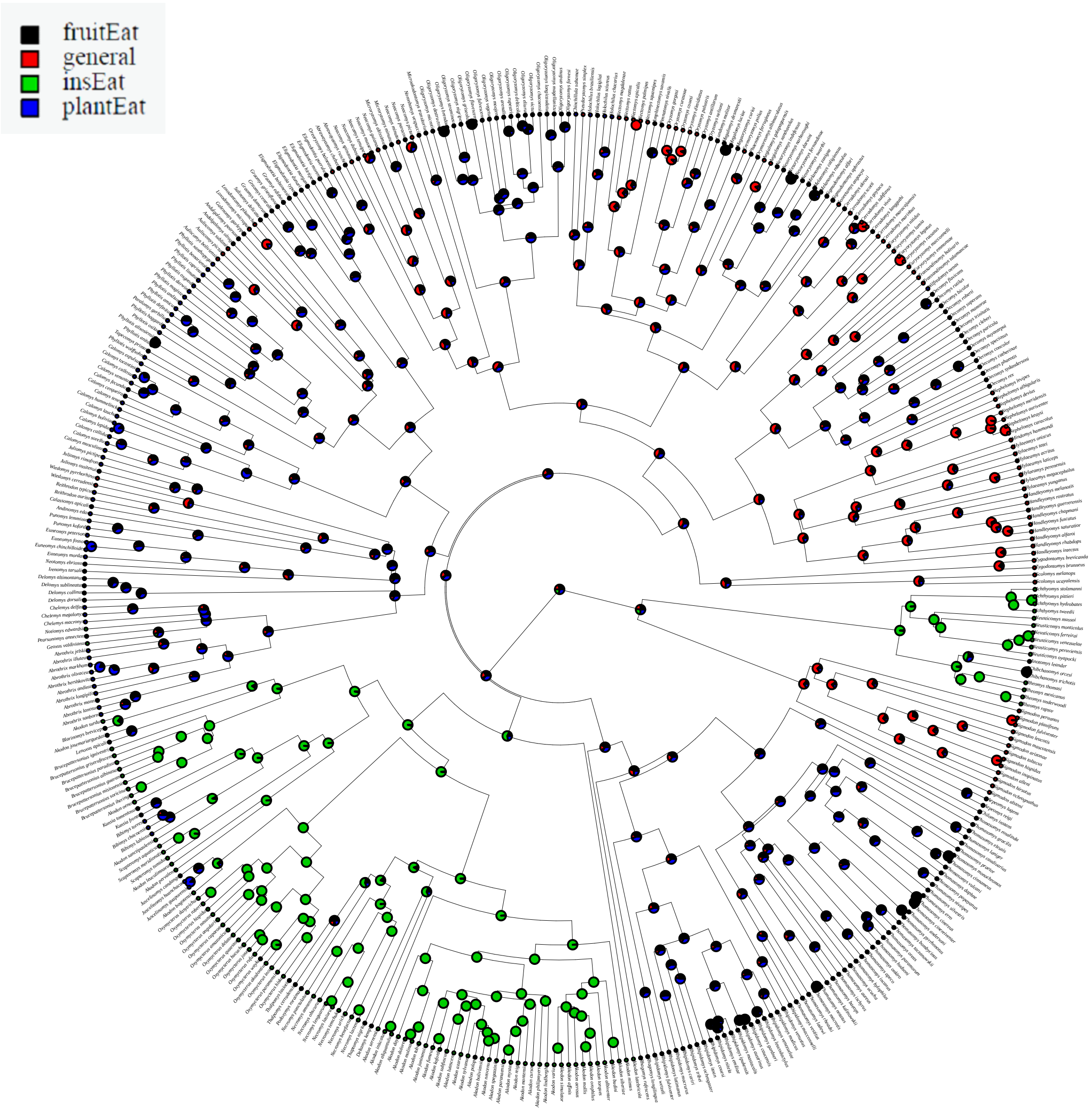
fruitEat
general
insEat
plantEat

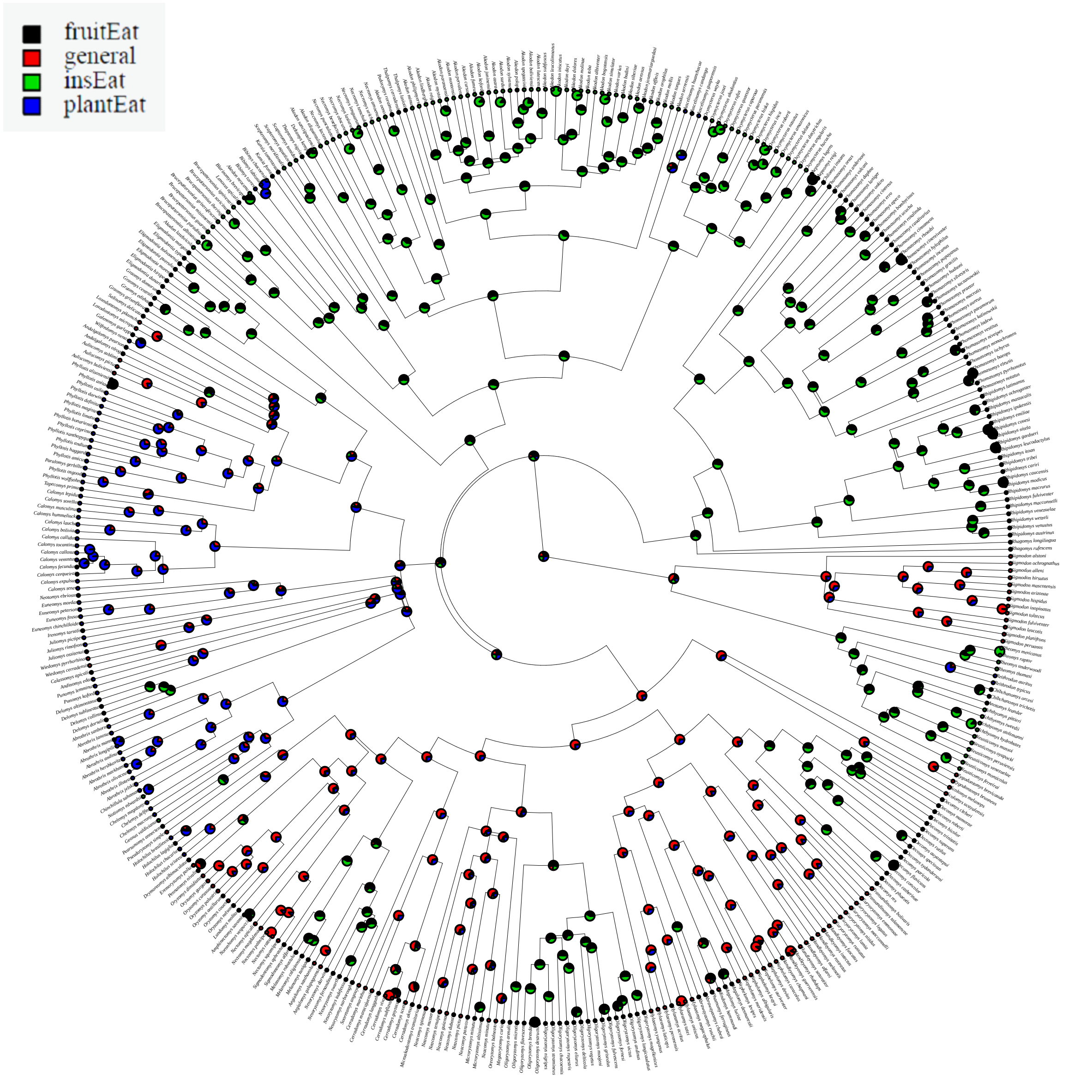
fruitEat
general
insEat
plantEat

Supplementary results

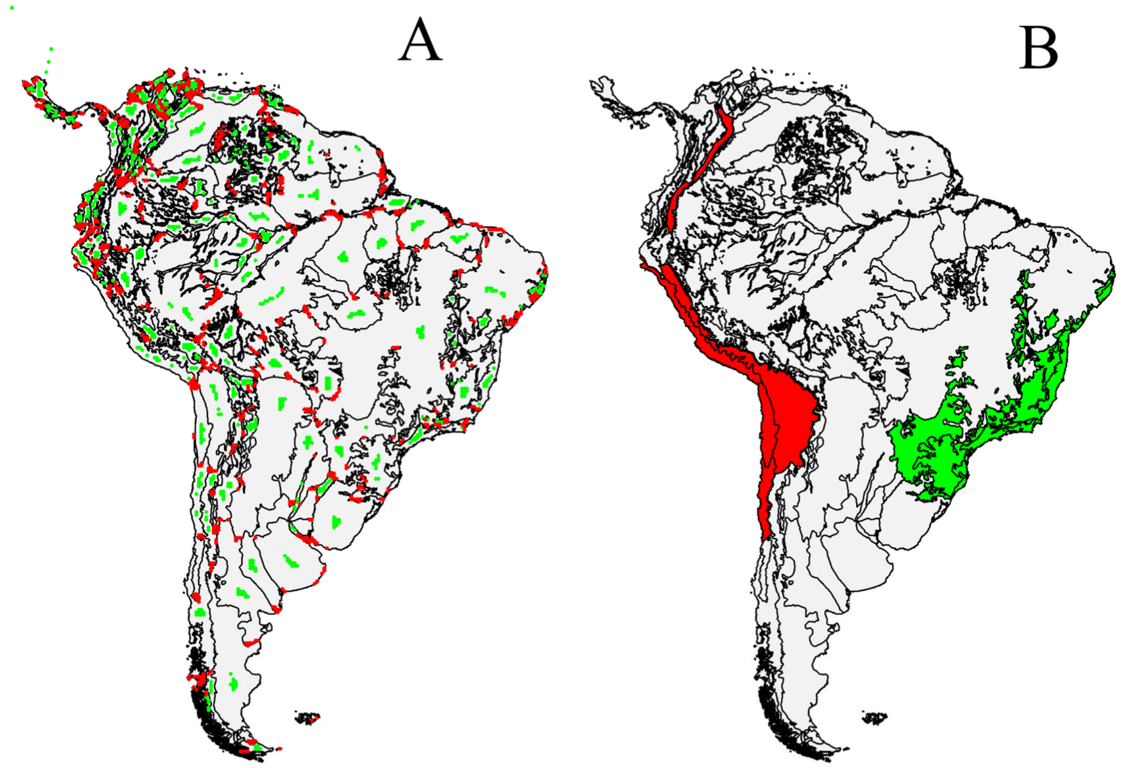

Fig. S1: Map of Neotropical ecoregions included in our study. In A we show the position of points within ecoregion cores (green) and ecotones (red). In B we show the position of Andean ecoregions (red) and those in Atlantic Rainforest (green); ecoregions belonging to other regions are in light gray. Maps in Lambert Equal-Area Projection.

Table S2: Comparison of linear mixed models to determine which spatial structure best fits the data. Exponential structure represents a model with exponential correlation structure. Exponential structure with nugget effect represents a model with exponential correlation structure and nugget effect. DF= degrees of freedom used by the LMM. ‘AIC’ is the Akaike Information Criterion. Akaike Information Criterion values were averaged across 2,000 models, a random subsample of a total of 10,000 estimates (100 ancestral character simulations for each of the 100 phylogenies). The best model across estimates was the one with lowest or most negative AIC values.

Assemblage –level tip-based metric	DF	AIC	Percentage of estimates as the best model
Transition rates (aTR)			
Exponential structure	12	-3901.72 ± 1001.56	45
Exponential structure with nugget effect	13	-4097.890± 1028.187	55
Stasis time (aST)			
Exponential structure	12	1656.923± 1177.983	43
Exponential structure with nugget effect	13	1458.295± 1185.857	57
Last transition time (aLT)			
Exponential structure	12	6641.046± 595.4198	46
Exponential structure with nugget effect	13	6464.488± 645.1829	54

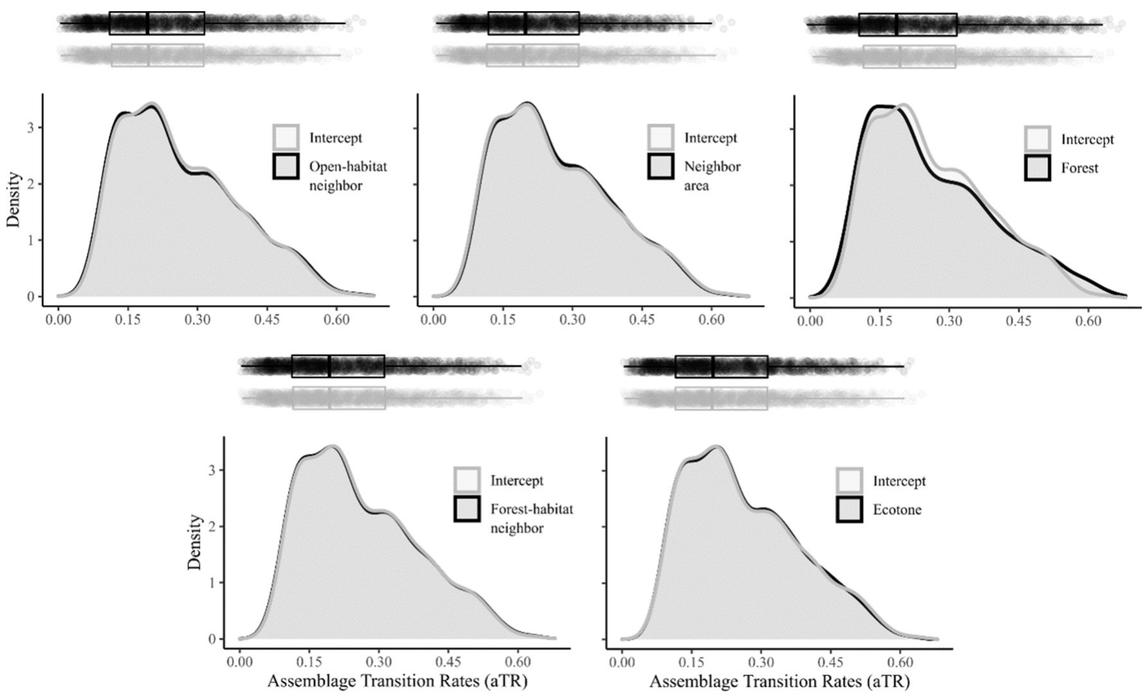

Fig. S2: Density plots of the intercept (expected mean) of assemblage transition rates aTR, and regression coefficient (deviation from the mean) of the least important variables. In each plot, the intercept is represented by the gray line and the regression coefficient is represented by the black line. Estimates were extracted from Linear Mixed Models that consider ecoregion-scale variables as fixed effects, ecoregion ID as random effect, and exponential correlation structure with nugget effect to accommodate spatial autocorrelation. Intercept and regression coefficients were extracted from each one of the 2,000 models. Boxplot in the upper margin shows average and 1st and 3rd quartiles of the distribution of aTR.

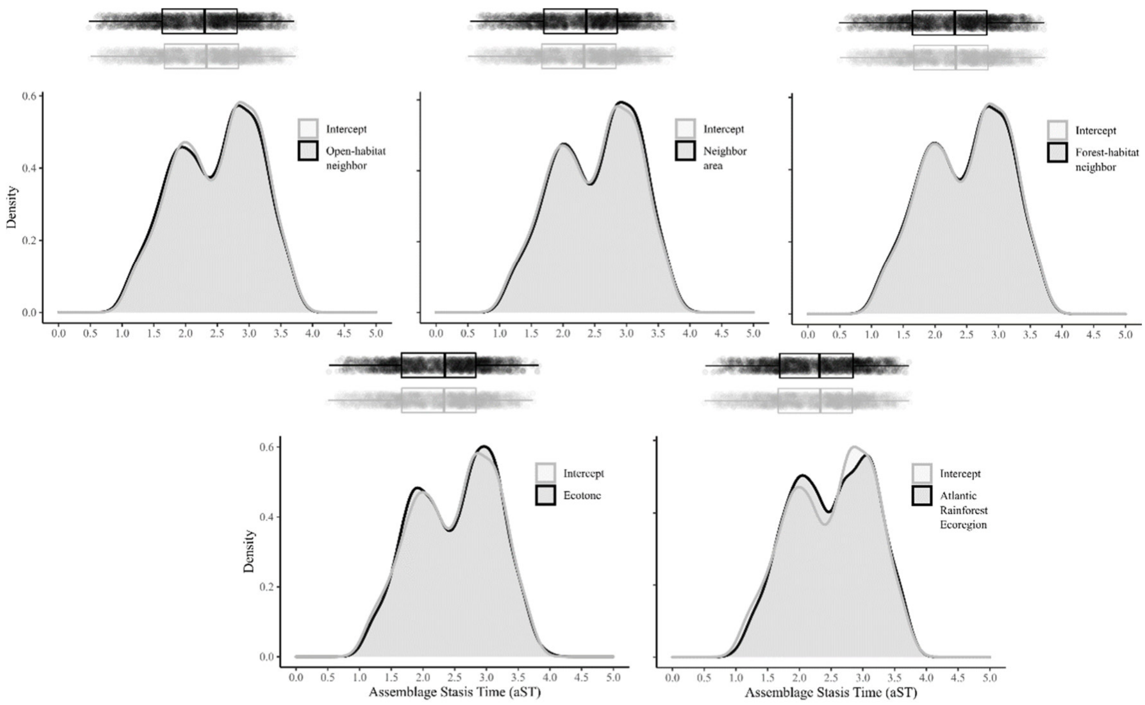

Fig. S3

Fig. S3: Density plots of the intercept (expected mean) of assemblage stasis time aST, and regression coefficient (deviation from the mean) of the least important variables. In each plot, the intercept is represented by the gray line and the regression coefficient is represented by the black line. Estimates were extracted from Linear Mixed Models that consider ecoregion-scale variables as fixed effects, ecoregion ID as random effect, and exponential correlation structure with nugget effect to accommodate spatial autocorrelation. Intercept and regression coefficients were extracted from each one of the 2,000 models. Boxplot in the upper margin shows average and 1st and 3rd quartiles of the distribution of aST.

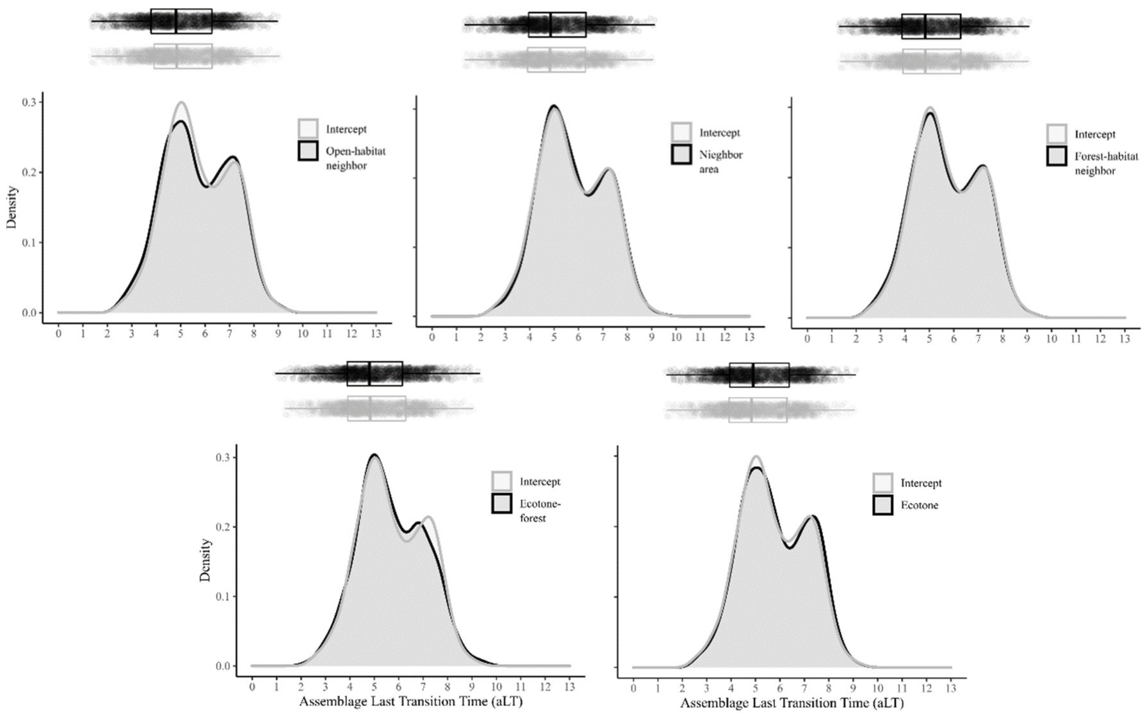

Fig. S4: Density plots of the intercept (expected mean) of assemblage last transition time aLT, and regression coefficient (deviation from the mean) of the least important variables. In each plot, the intercept is represented by the gray line and the regression coefficient is represented by the black line. Estimates were extracted from Linear Mixed Models that consider ecoregion-scale variables as fixed effects, ecoregion ID as random effect, and exponential correlation structure with nugget effect to accommodate spatial autocorrelation. Intercept and regression coefficients were extracted from each one of the 2,000 models. Boxplot in the upper margin shows average and 1st and 3rd quartiles of the distribution of aLT.

Supplementary results considering small-ranged species

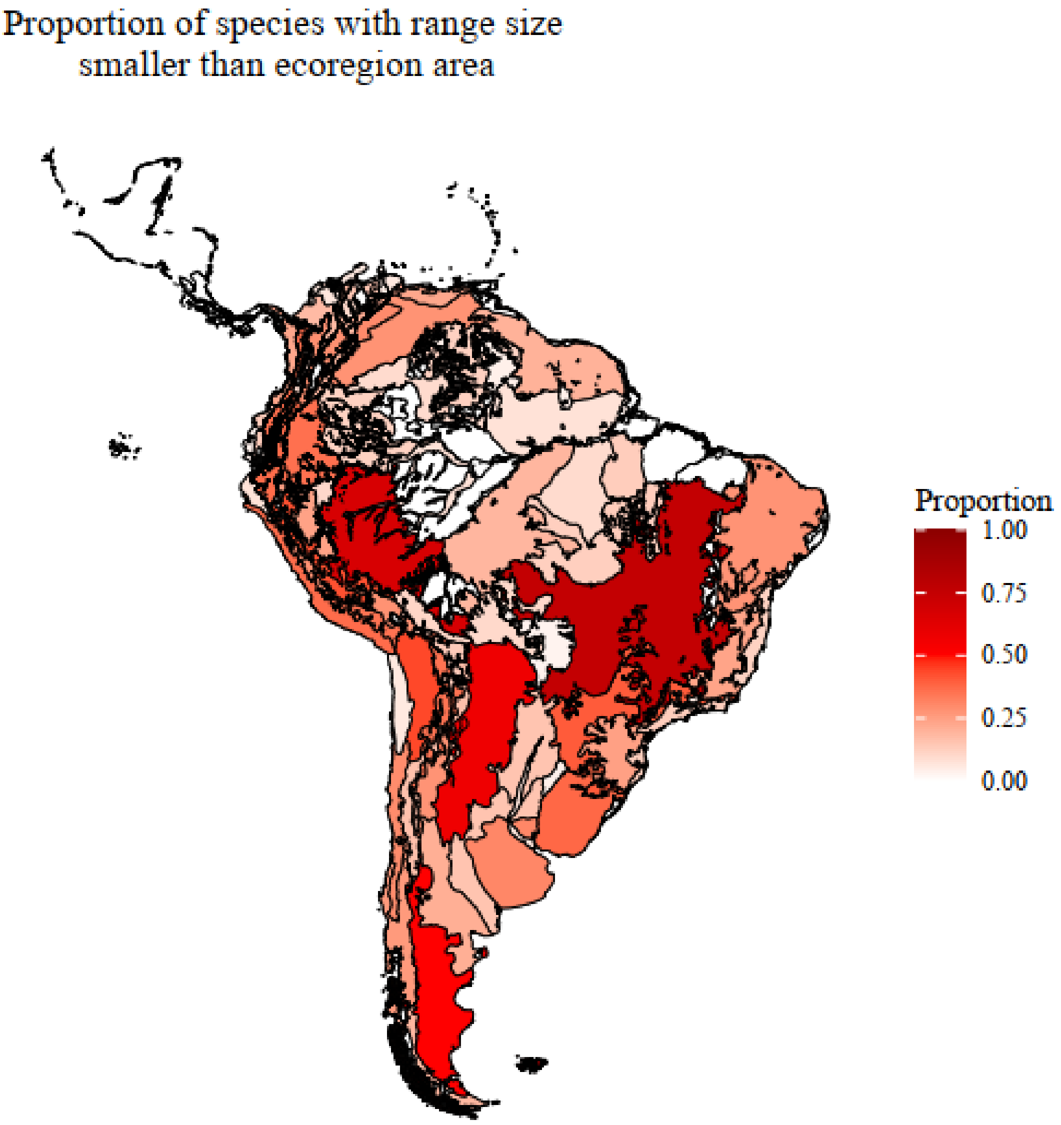

Fig. S5: Proportion of sigmodontine rodent species with range size smaller than ecoregion area. Map in Lambert Equal-Area projection.

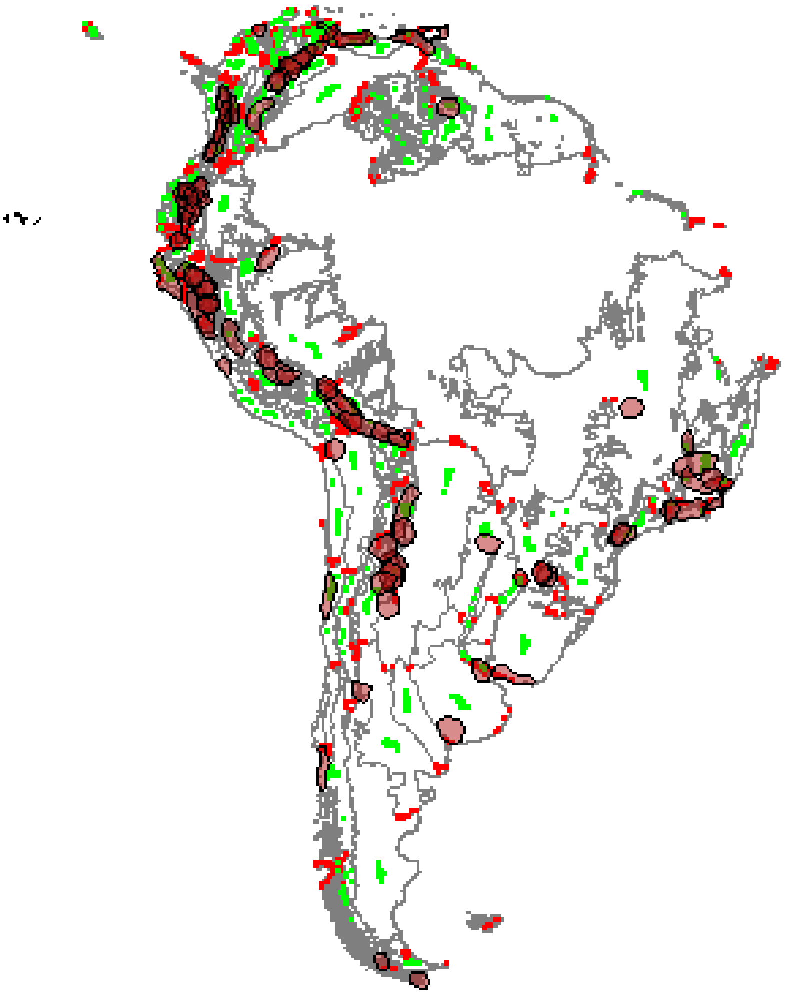

Fig. S6: Distribution of small-ranged species of sigmodontine rodents (red polygons). Species shown here have a range smaller than ecoregion area. In the background we show points located in ecoregion core (green points) and ecotone (red points). Map in Lambert Equal-Area projection.

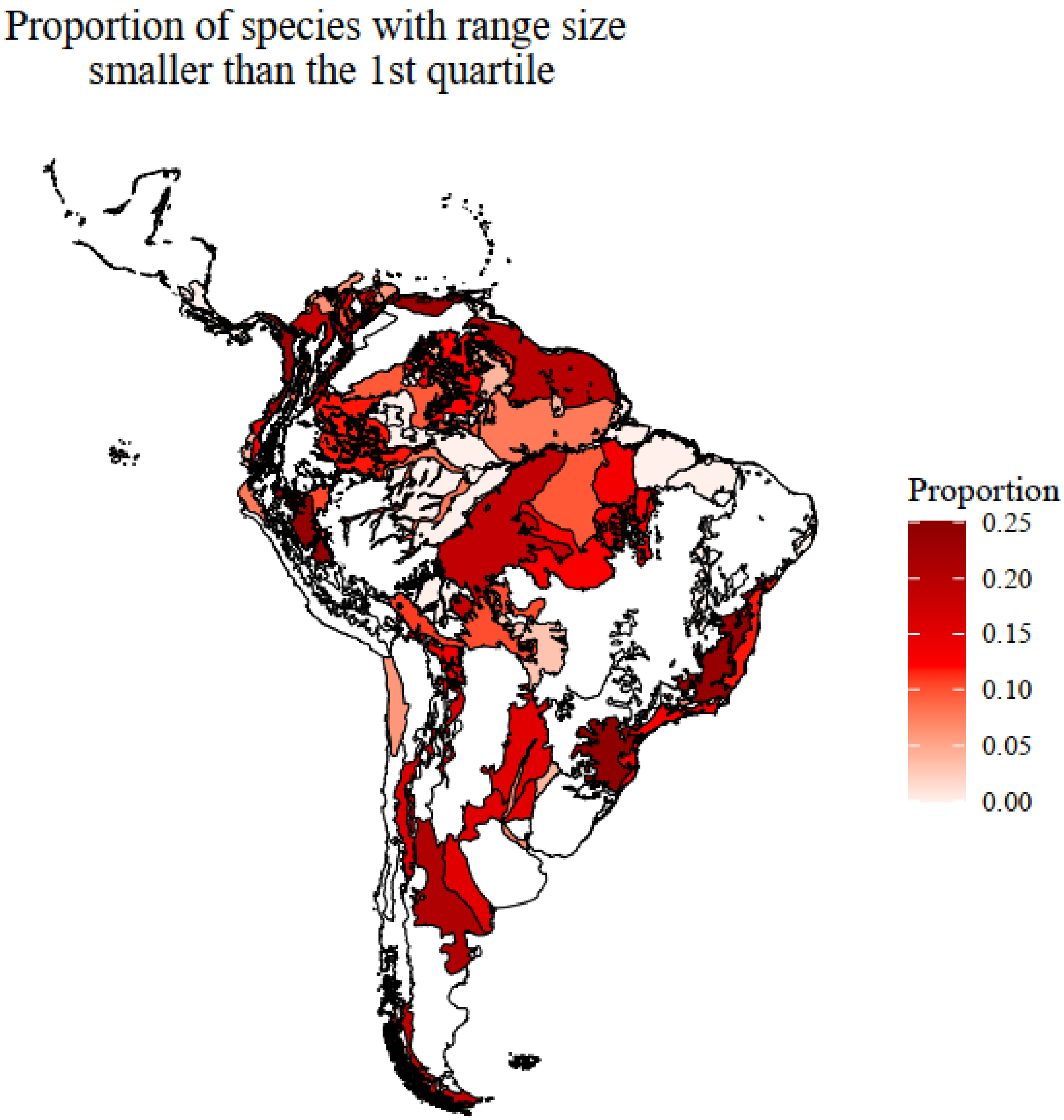

Fig. S7: Number of small-ranged species of sigmodontine rodents per ecoregion. We considered as small-ranged species those having a range size smaller than 4.16 square degrees, which is the value of the 1st quartile of the distribution of range-size values across the 350 sigmodontine species included in our dataset. Map in Lambert Equal-Area projection.

Table S3: Comparison of linear mixed models to determine which spatial structure best fits the data of small-ranged species. Exponential structure represents a model with exponential correlation structure. Exponential structure with nugget effect represents a model with exponential correlation structure and nugget effect. DF= degrees of freedom used by the LMM. ‘AIC’ is the Akaike Information Criterion. Akaike Information Criterion values were averaged across 2,000 models, a random subsample of a total of 10,000 estimates (100 ancestral character simulations for each of the 100 phylogenies). The best model across estimates was the one with lowest or most negative AIC values.

Assemblage –level tip-based metric	DF	AIC	Percentage of estimates as the best model
Transition rates (aTR)			
Exponential structure	12	-567.05± 153.85	77.2
Exponential structure with nugget effect	13	-571.82± 178.53	22.8
Stasis time (aST)			
Exponential structure	12	359.56±197.43	78
Exponential structure with nugget effect	13	356.62 ± 197.95	22
Last transition time (aLT)			
Exponential structure	12	1081.72 ± 125.04	50
Exponential structure with nugget effect	13	1076.46 ± 121.79	50

Table S4: Average parameter value ± standard deviation representing phylogenetic uncertainty on estimates of fixed effects, random effect, and spatial correlation structure, across the 2,000 linear mixed models, and considering the data set of small-ranged species. Fixed effects are represented in standard deviations from the intercept for each assemblage-level tip-based metric (columns).

Effect/ variable	Transition rates	Stasis time	Last transition times
	Average estimates ± standard deviation		
Fixed effect			
Intercept	0.253± 0.156	2.517± 0.778	6.515± 2.256
Position	0.004± 0.021	0.053± 0.140	0.047± 0.477
Habitat type	0.010± 0.028	-0.023± 0.205	-0.052± 0.775
Interaction position x habitat	-0.005± 0.031	-0.057± 0.179	0.019± 0.762
Sum of neighbor's area	-0.002± 0.010	-0.040± 0.071	-0.069± 0.085
Point overlap with forest-habitat ecoregions	0.001± 0.008	0.030± 0.058	0.072± 0.060
Point overlap with open-habitat ecoregions	0.002± 0.010	0.013± 0.064	-0.013± 0.087
Atlantic Rainforest ecoregions	-0.002± 0.067	0.079± 0.276	-0.695± 0.266
Andean ecoregions	-0.009± 0.037	-0.078± 0.208	-0.173± 0.391
Random effect			
Standard deviation (σ)	0.029 ± 0.033	0.080± 0.139	0.651± 0.781
Residual	0.114± 0.034	0.752± 0.246	3.154± 0.707
Spatial correlation structure			
Range (*r*)	2.585299e+264± 2.25157e+52	2.252003e+277± 6.821561e+90	2.295349e+235± 89.15
Nugget (*n*)	-	-	-

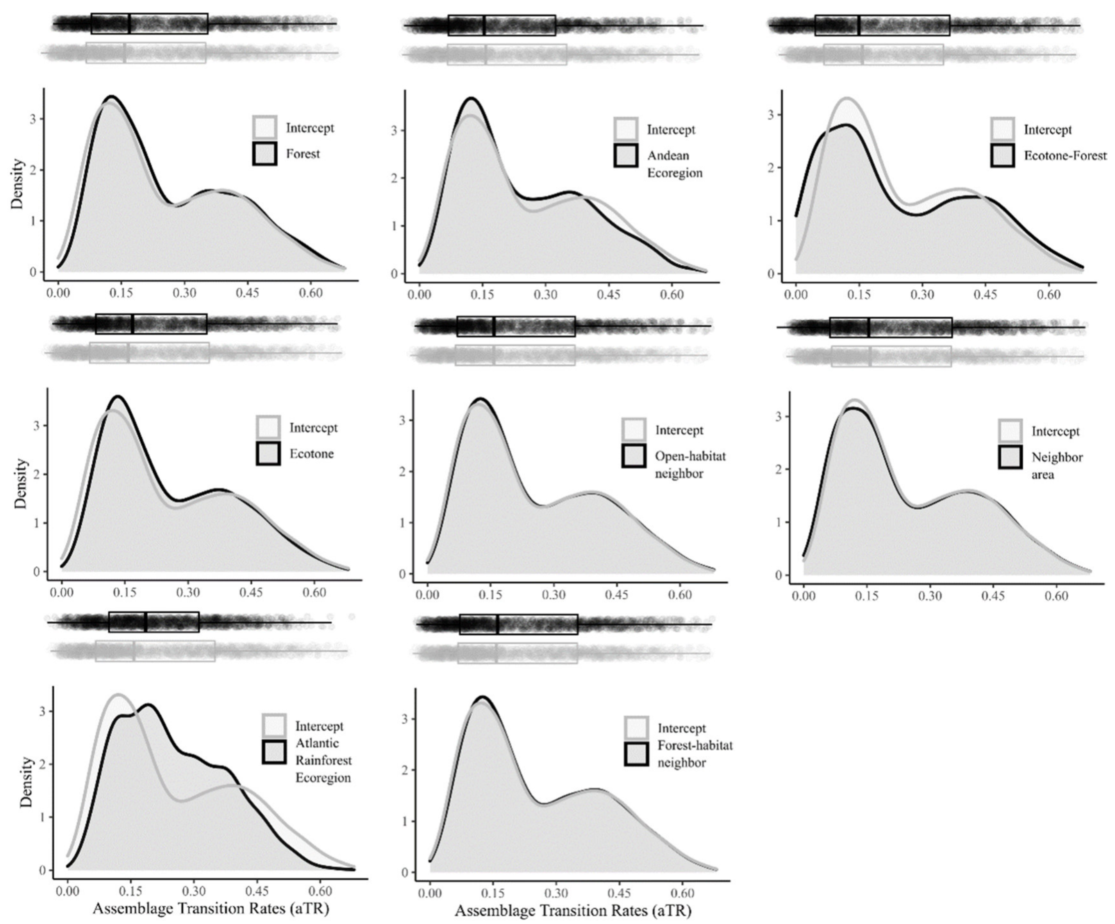

Fig. S8: Density plots of the intercept (expected mean) of assemblage last transition time aTR, and regression coefficient (deviation from the mean) of the most important variables considering small-ranged species. In each plot, the intercept is represented by the gray line and the regression coefficient is represented by the black line. Estimates were extracted from Linear Mixed Models that consider ecoregion-scale variables as fixed effects, ecoregion ID as random effect, and exponential correlation structure without nugget effect to accommodate spatial autocorrelation. Intercept and regression coefficients were extracted from each one of the 2,000 models. Boxplot in the upper margin shows average and 1st and 3rd quartiles of the distribution of aTR.

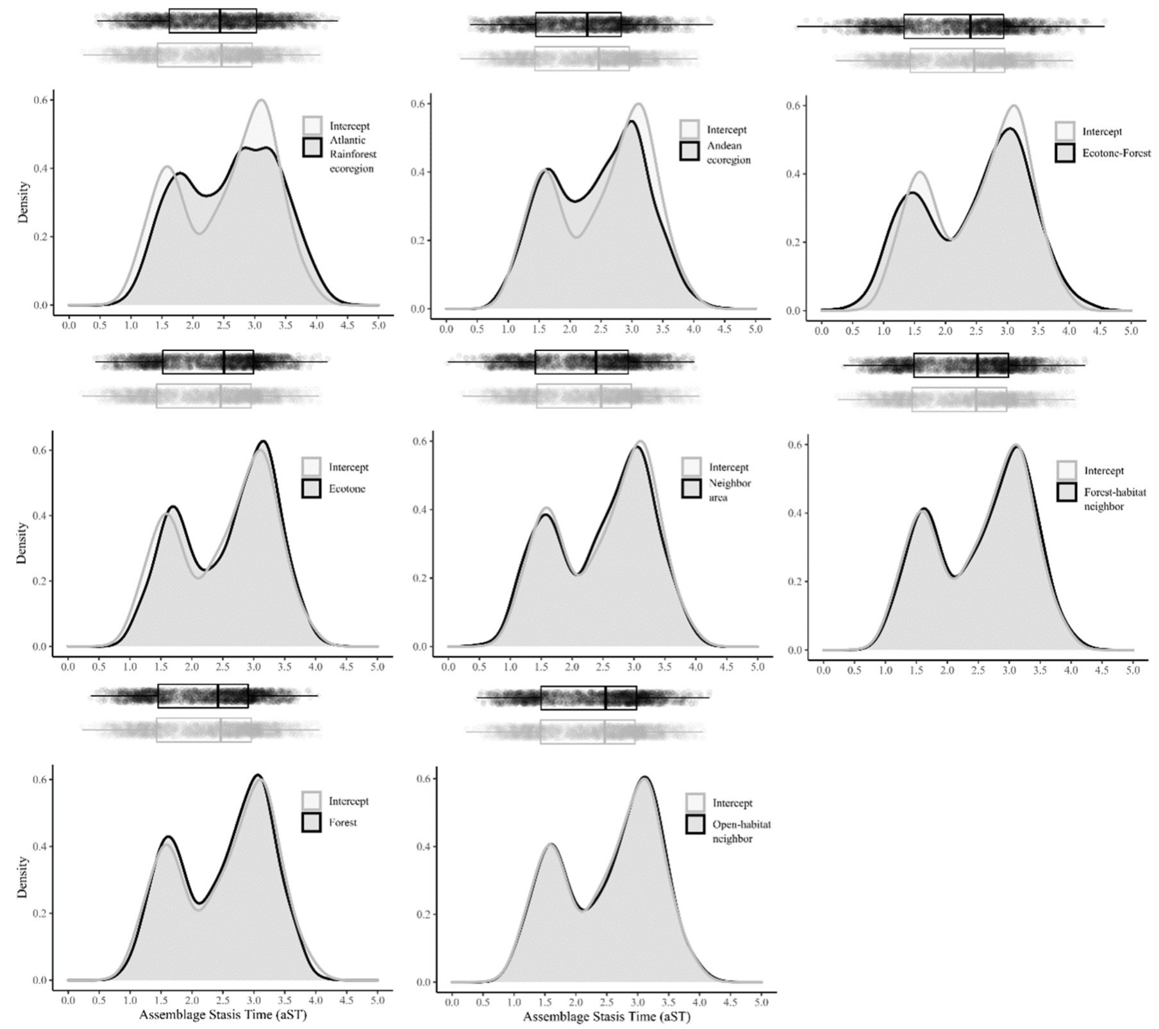

Fig. S9: Density plots of the intercept (expected mean) of assemblage last transition time aST, and regression coefficient (deviation from the mean) of the most important variables considering small-ranged species. In each plot, the intercept is represented by the gray line and the regression coefficient is represented by the black line. Estimates were extracted from Linear Mixed Models that consider ecoregion-scale variables as fixed effects, ecoregion ID as random effect, and exponential correlation structure without nugget effect to accommodate spatial autocorrelation. Intercept and regression coefficients were extracted from each one of the 2,000 models. Boxplot in the upper margin shows average and 1st and 3rd quartiles of the distribution of aST.

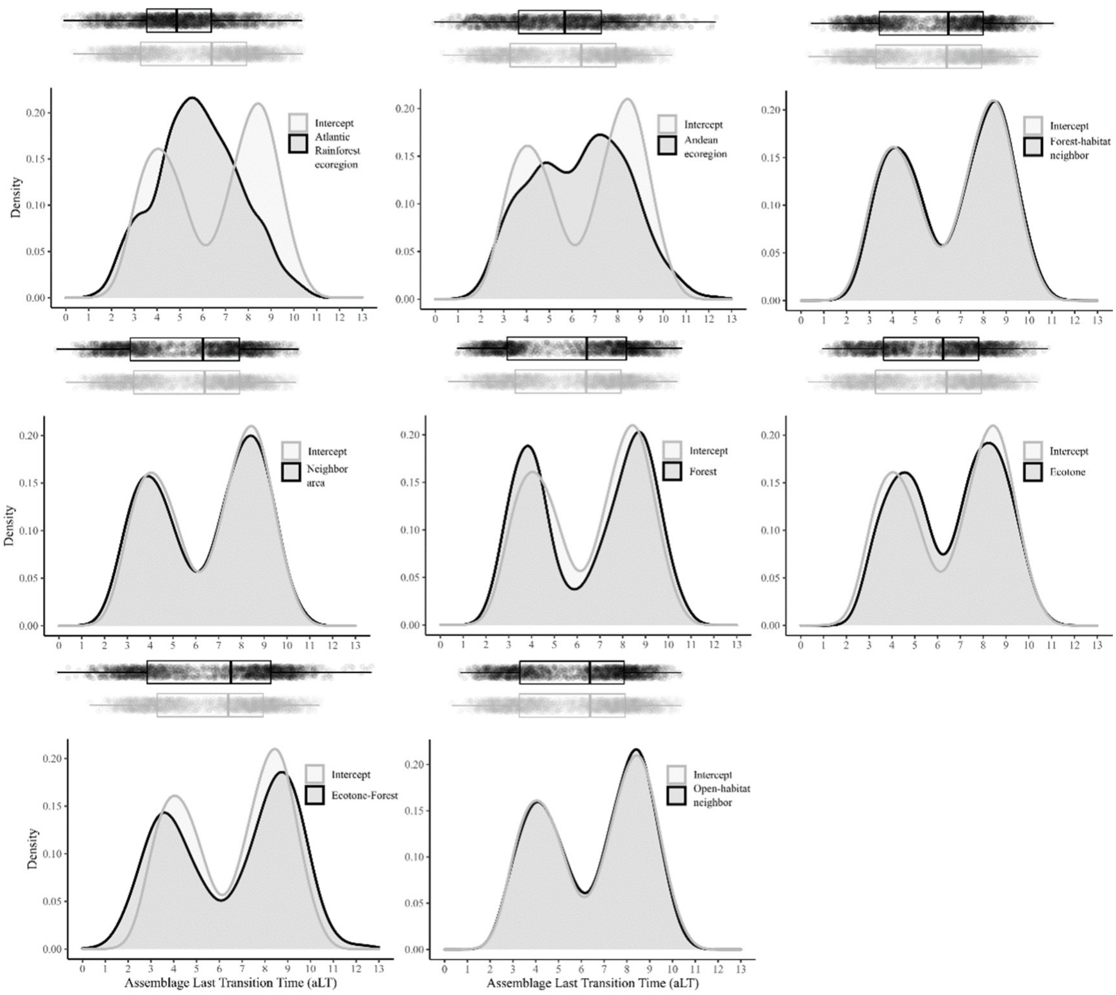

Fig. S10: Density plots of the intercept (expected mean) of assemblage last transition time aLT, and regression coefficient (deviation from the mean) of the most important variables considering small-ranged species. In each plot, the intercept is represented by the gray line and the regression coefficient is represented by the black line. Estimates were extracted from Linear Mixed Models that consider ecoregion-scale variables as fixed effects, ecoregion ID as random effect, and exponential correlation structure with nugget effect to accommodate spatial autocorrelation. Intercept and regression coefficients were extracted from each one of the 2,000 models. Boxplot in the upper margin shows average and 1st and 3rd quartiles of the distribution of aLT.

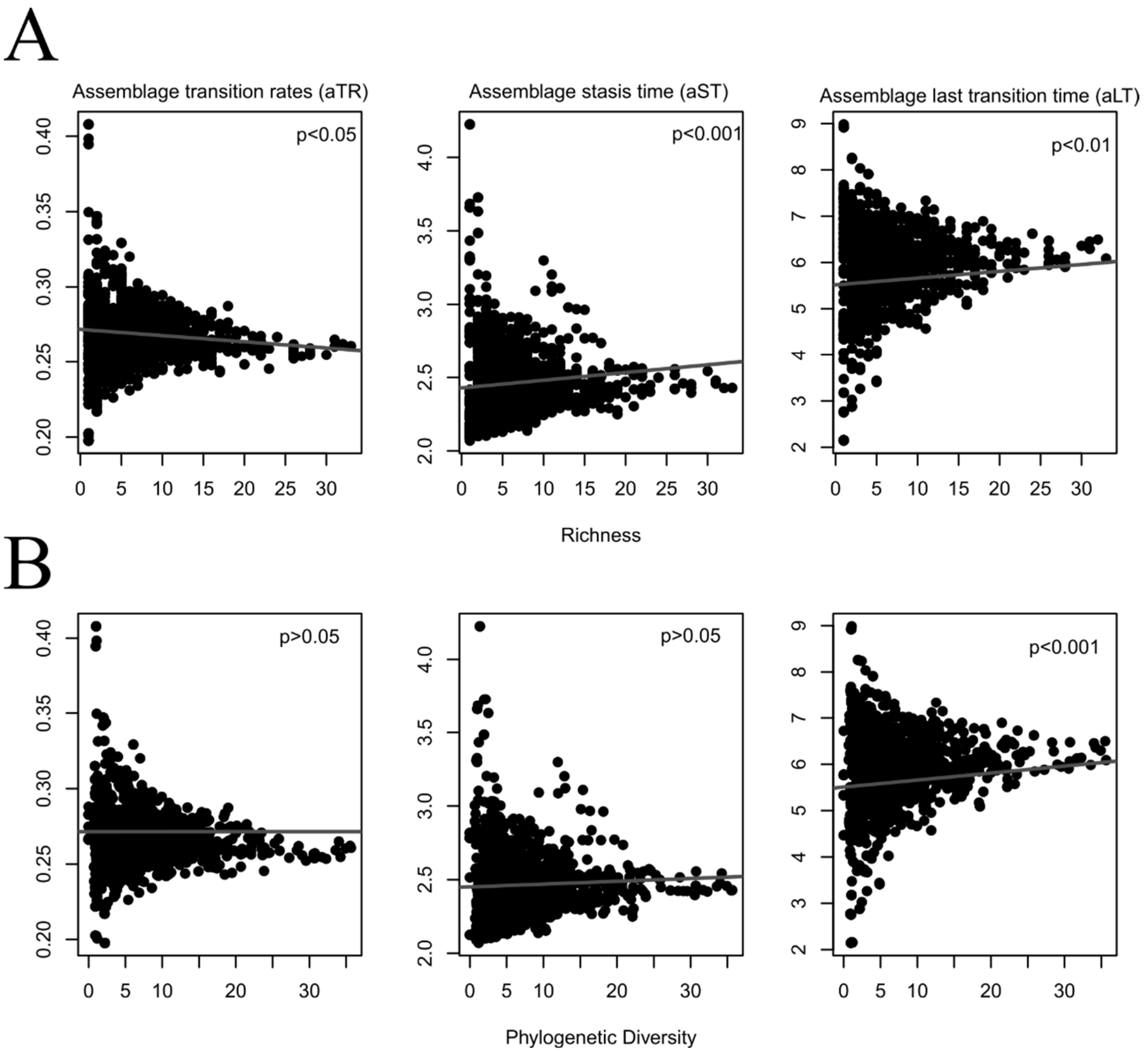

Fig. S11: Relationship between assemblage-level tip-based metrics and species richness (A) and phylogenetic diversity (B). P-values and regression slopes were produced by generalized least squares regressions that tested the relationships while accounting for spatial autocorrelation. The unit of stasis time and last transition time is in millions of years.

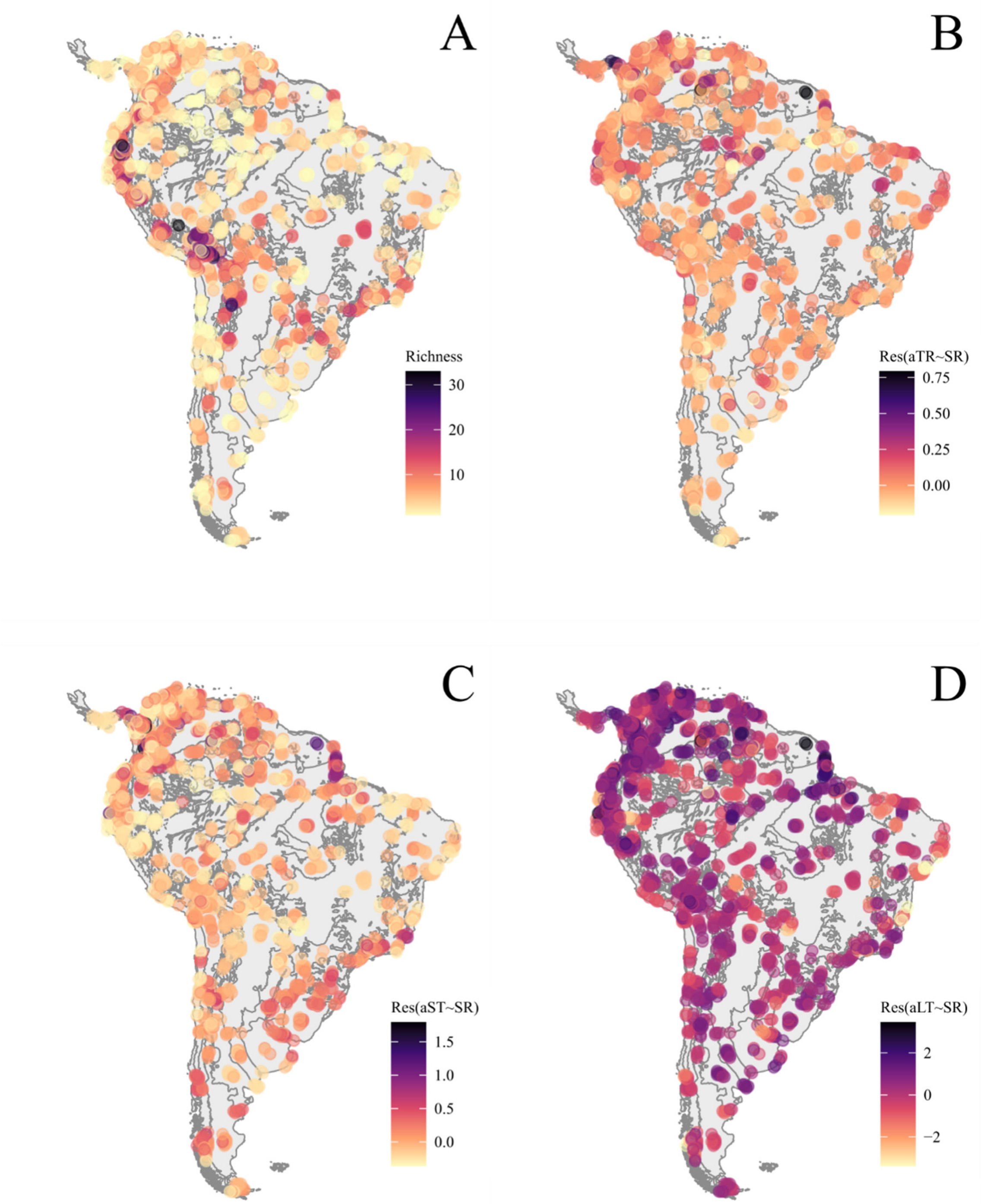

Fig. S12: Mapped species richness (SR, A) and residuals of generalized least squares regressions between assemblage-level transition rates (aTR, B), stasis time (aST, C), last transition time (aLT, D) and species richness (SR) of sigmodontine rodents at core and ecotones of Neotropical ecoregions. Maps in Lambert Equal-Area projection.

Table S5: Pearson's linear correlation between the assemblage-level tip-based metrics. These correlations were based on averaged values of assemblage-level tip-based metric across the 10,000 estimates. P-values are presented below the diagonal.

	aTR	aST	aLT
aTR	1	0.36	-0.23
aST	< 2.2e-16	1	0.46
aLT	< 2.2e-16	< 2.2e-16	1

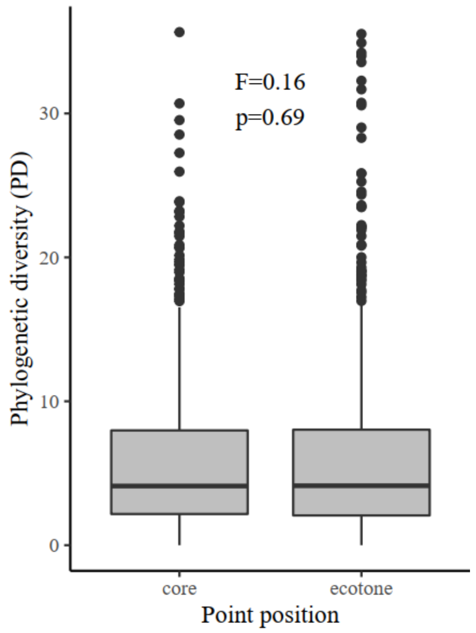

Fig. S13: Variation in phylogenetic diversity (the sum of phylogeny branch lengths connections species of a community) relative to point position in ecoregion core and ecotone.
